# Synthesis and Characterization of Furan-Based Methacrylate Oligomers Containing the Imine Functional Group for Stereolithography

**DOI:** 10.1021/acsomega.4c03274

**Published:** 2024-07-04

**Authors:** Nuttapol Risangud, Jittima Mama, Piyarat Sungkhaphan, Puttipong Pananusorn, Orawan Termkunanon, Muhammad Sulthan Arkana, Supang Sripraphot, Tareerat Lertwimol, Somprasong Thongkham

**Affiliations:** † National Metal and Materials Technology Center, National Science and Technology Development Agency, 111 Thailand Science Park, Phahonyothin Road, Klong Luang, Pathum Thani 12120, Thailand; ‡ National Nanotechnology Center, National Science and Technology Development Agency, 111 Thailand Science Park, Paholyothin Road, Klong 1, Klong Luang, Pathumthani 12120, Thailand; § Petroleum and Petrochemical College, Chulalongkorn University, Bangkok 10330, Thailand; ∥ Department of Materials Science and Engineering, School of Molecular Science and Engineering, Vidyasirimedhi Institute of Science and Technology (VISTEC), Wangchan, Rayong 21210, Thailand

## Abstract

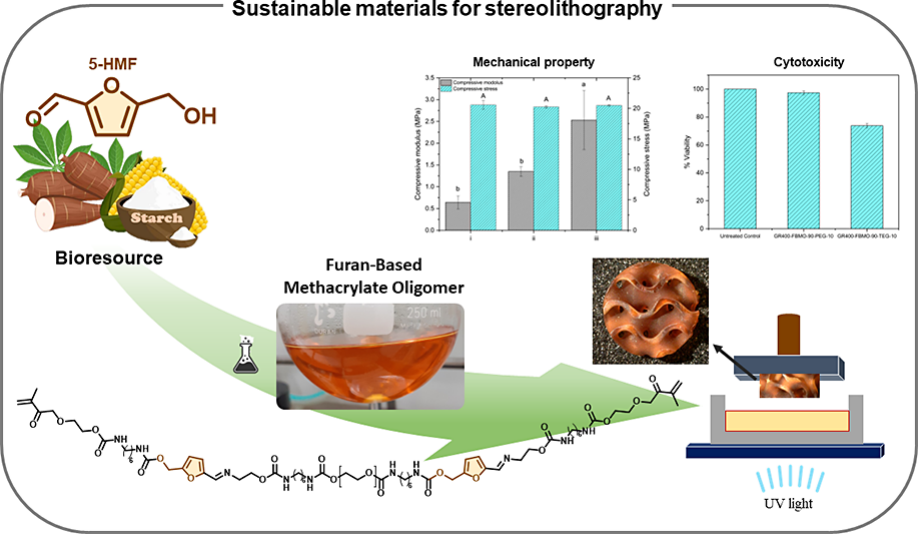

Herein, a furan-based methacrylate oligomer (FBMO) featuring imine functional groups was synthesized for application in stereolithography. The preparation involved the imination reaction of 5-hydroxymethylfurfural (5-HMF) and amino ethanol. Utilizing 5-HMF as a sustainable building block for furan-based polymers, FBMO was formulated and subsequently integrated into photosensitive resin formulations along with methacrylate-containing diluents, such as PEGDMA and TEGDMA. The synthesized furan-based methacrylate oligomers underwent comprehensive characterization using FTIR, ^1^H NMR spectroscopy, and size exclusion chromatography. The impact of methacrylate-containing diluents on various properties of the formulated resins and the resulting 3D-printed specimens was systematically evaluated. This assessment included an analysis of rheological behavior, printing fidelity, mechanical properties, thermal stability, surface morphology, and cytotoxicity. By adjusting the ratios of FBMO to methacrylate-containing diluents within the range of 50:50 to 90:10, the viscosity of the resulting resins was controlled to fall within 0.04 to 0.28 Pa s at a shear rate of 10 s^–1^. The 3D-printed specimens exhibited precise conformity to the computer-aided design (CAD) model and demonstrated compressive moduli ranging from 0.53 ± 0.04 to 144 ± 6.70 MPa, dependent on the resin formulation and internal structure. Furthermore, cytotoxicity assessments revealed that the 3D-printed specimens were noncytotoxic to porcine chondrocytes. In conclusion, we introduce a new strategy to prepare the furan-based methacrylate oligomer (FBMO) and 3D-printed specimens with adjustable properties using stereolithography, which can be further utilized for appropriate applications.

## 1 Introduction

Most plastics are derived from petroleum-based sources, and the associated production process is marked by environmental pollution through the release of carbon dioxide (CO_2_), a significant contributor to global warming. Therefore, the utilization of biobased materials appears to be a more sustainable alternative to replace petroleum-based counterparts. Within the realm of biobased platform chemicals, 5-hydroxymethylfurfural (5-HMF) stands out as a green potential building block for the synthesis of furan-based polymers, such as poly(ethylene 2,5-furandicarboxylate) derived from 2,5-furandicarboxylic acid (FDCA).^1−3^

5-HMF has increasingly emerged as a prominent candidate for a primary value-added chemical in the fields of industrial chemistry and polymer science.^4^ Its applications extend to various chemical precursors, including FDCA, levulinic acid (LA), 5-ethoxymethylfurfural (5-EMF), 2,5-dimethylfuran (DMF), and 2,5-bis(hydroxymethyl)furan (BHMF).^5−10^ Furthermore, 5-HMF finds utility in diverse applications, that is, wood composites,^11^ cross-linked polymers, fuel additives, and fiber glass composites.^12,13^ To the best of our knowledge, there have been a research work reported in the literature related to the utilization of 5-HMF in stereolithography resin compositions.^14^ A series of bioderived furanic (meth)acrylates as reactive diluents were reported. The furanic diluents can reduce the viscosity of commercial stereolithography resins, participate in photocuring with high conversion of polymerizable groups, and satisfy performance requirements. From the perspective of biobased resin in UV-curing additive manufacturing, a research study has detailed the development of a series of biobased polyester resins sourced from itaconic acid.^15^ These novel resins, comprising 56 to 100% biobased content, were studied for the first time to understand how variations in their chemical structures, such as double bond density, flexibility, and aromatic content, affect their physicochemical properties. The results revealed that these materials provide customizable characteristics suitable for use in additive manufacturing, with a maximum biobased content of 85%.

Numerous studies focusing on the application of 5-hydroxymethylfurfural (5-HMF) in polyurethane have been documented. For instance, Xiao-Jing Li et al. undertook the fabrication of high molecular weight polyurethanes (PU) incorporating furan-based nonisocyanate structures.^16^ The furan-based nonisocyanate polyurethanes (NIPUs) were synthesized through a four-step synthesis process including reduction, glycidylation, carbonation of CO_2_, and step-growth polymerization with diamines. These NIPUs were then combined with poly(propylene carbonate) (PPC) to enhance the thermal and mechanical properties of the composite material. In a separate study, Olivito et al. detailed the preparation of biobased and biodegradable polyether/polyurethane (PU) foam by reacting 2,5-bis(hydroxymethyl)furan (BHMF) with a prepolymer.^17^ Recently, Cheng et al. contributed to the field by reporting the synthesis of two new furan-based acrylate monomers, namely, 2-hydroxyethyl 2-(furan-2-yl(hydroxy)methyl)acrylate and methyl 2-(hydroxy(5-(hydroxymethyl)furan-2-yl)methyl)acrylate. These monomers were synthesized through the Baylis-Hillman reaction of 5-HMF and acrylate, serving as crucial components for the preparation of novel healable polyurethane (PU).^18^

The 3D printing technology to fabricate predesigned and high-fidelity architectural objects through a layer-by-layer process through computer-aided design (CAD) of polyurethane offers excellent opportunities to prepare a wide range of specimens with desired physicochemical and mechanical properties.^19^ The unique properties of polyurethane-elastomer hold great potential for multiple applications. Nonetheless, polyurethane-based 3D-printed specimens have primarily been fabricated from petroleum-derived polymers.^20,21^ In recent years, biobased polymers, including polyurethane, have emerged as an alternative material for various applications. The main advantages of bioderived materials compared to conventional polymers are biodegradability, ease of recovery, and carbon neutrality^22^ Combining the mechanical properties of polyurethane-elastomer, 3D-printing technology, and biobased polymer materials has become an exciting topic for fabricating unique 3D-printed objects. Among 3D printing technology, light-assisted additive manufacturing (AM) techniques enable greater control of the dimensional accuracy of specimens because a liquid photopolymer is solidified by light-activated polymerization. This vat photopolymerization offers efficient, rapid, and feasible prototyping at ambient temperature.^23,24^

Printing materials for light-assisted AM techniques, frequently referred to as photosensitive resin, require photocurable moieties commonly composed of multifunctional (meth)acrylate or epoxy monomers. Several research groups have developed powerful strategies to address the limitation of petroleum-derived photopolymers by replacing those with bioderived materials to formulate 3D-printing materials.^25−28^ Recently, Hu et al. introduced biobased polyurethane photopolymers for digital light processing (DLP) from rubber seed oil (RSO).^29^ The RSO-based polyurethane photopolymers were prepared through the alcoholysis reaction RSO with glycerol; subsequently, the resultant polymer was reacted with isophorone diisocyanate (IPDI) and hydroxyethyl acrylate (HEA), achieving the acrylate-containing RSO. The formulated resin was the fabricated football-ene model through DLP, and the obtained specimen was apparently well accordant with the CAD model and revealed a reasonably high mechanical strength with approximately 16.3 MPa. However, the cytotoxicity test of printed samples has not been investigated. Besides, other photosensitive resins prepared from biobased photopolymer, not only polyurethane-based resin, have been intensively introduced for utilizing new sustainable specimens through stereolithographic 3D-printing methods. To the best of our knowledge, the utilization of 5-HMF in biobased photosensitive resins for light-assisted AM has not yet been reported.

This work introduces furan-based methacrylate oligomers (FBMO) containing an imine functional group exploited for stereolithography. 5-HMF was employed to prepare FBMO, which was subsequently prepared for the light-assisted printing materials. The effect of the methacrylate-containing diluents in the resins on the properties of formulated resins and 3D-printed specimens, for example, rheological behavior, printing fidelity, mechanical property, surface morphology, and cytotoxicity, were assessed. Besides, we also introduce the strategy to fine-tune the mechanical performance of 3D-printed constructs fabricated through a light-assisted 3D printer by varying their internal structure. The cytotoxicity result revealed that the 3D-printed specimens were noncytotoxic to porcine chondrocytes. This 5-HMF-based methacrylate oligomer containing imine functional groups shows great potential for stereolithography printing resins in different applications.

## 2 Materials and Methods

### 2.1 Materials

Chloroform (AR) was obtained from RCI labscan, and ethanolamine with purity >99%, hexamethylene diisocyanate (HDI) with purity >98% and poly(ethylene glycol) (PEG1000, *M*_n_ 1000 Da) were purchased from Tokyo Chemical Industry Co., Ltd. (TCI). 2-Hydroxyethyl methacrylate (HEMA) with purity 97% was purchased from S.M. Chemical Supplies, 5-hydroxymethylfurfural (5-HMF), and tetrahydrofuran (THF) for analysis were obtained from Carlo Erba reagents. Triethylene glycol dimethacrylate (TEGDMA), poly(ethylene glycol dimethacrylate) (PEGDMA, *M*_n_ = 750 Da), diphenyl (2, 4, 6-trimethylbezoyl) phosphine oxide (TPO, photoinitiator), 3-[4,5-dimethylthiazol-2-yl]-2,5 diphenyl tetrazolium bromide (MTT), and collagenase I were obtained from Sigma-Aldrich. Hydroquinone (inhibitor) was purchased from Fluka. Dichloromethane, isopropyl alcohol, and hexane were obtained from Qchemical. Dulbecco’s modified Eagles’ medium (DMEM), Dulbecco’phosphate-buffered saline (DPBS), fetal bovine serum (FBS), 0.25% trypsin- EDTA solution, and nonessential amino acids solution (NEAA, 100X) were obtained from Gibco. Penicillin–streptomycin solution (P–S, 100 units/mL penicillin and 100 μg/mL streptomycin) was purchased from Corning.

### 2.2 FBMO Synthesis

#### 2.2.1 Preparation of Monomer N2

Ethanolamine (2.69 g, 0.044 mol) was introduced into a round-bottom flask under a nitrogen gas atmosphere and stirred for 15 min. Subsequently, 5-HMF (5.00 g, 0.040 mol) was dissolved in 5 mL of chloroform and added to the flask containing ethanolamine. The resulting mixture was stirred at room temperature for 1 h. The chloroform was then evaporated, yielding a dark brown liquid as the final product. Monomer N2 was characterized by FTIR and ^1^H NMR. FTIR: *v* = 3433 (O–H), 2850 (C–H), 1641, (C=N), 1020 (C–N). ^1^H NMR (δ, DMSO-*d*_6_, 400 MHz, ppm): 8.06 (s, 1H,–CHN−), 6.81, 6.82 and 6.41, 6.40 (d, 2H,–CHC−), 4.42 (s, 2H,–CH_2_OH), 3.60–3.53 (m, 4H,–N(CH_2_)_2_OH).

#### 2.2.2 Preparation of FBMO

PEG1000 (12 g, 0.012 mol) was heated to 60 °C for 20 min. Concurrently, HDI (12.12 g, 0.072 mol) was dissolved in 50 mL of THF. The solution of HDI was gradually added to the flask containing PEG1000, and the mixture was stirred continuously for 10 min. Subsequently, a solution of Monomer N2 (6.09 g, 0.036 mol) in THF (140 mL) was slowly introduced and stirred for 40 min. In the next step, HEMA (4.68 g, 0.036 mol) and Hydroquinone (3.6 mg, 0.033 mmol) were combined in THF (40 mL). The resulting mixture of HEMA solution was gradually added to the flask and stirred continuously for an additional 40 min. Finally, the product was filtered, and THF was evaporated. The ultimate outcome is a red-brown liquid. The resultant FBMO (95% yield) was characterized by FTIR, ^1^H NMR, and SEC. FTIR: *v* = 3336 (N–H), 2847 and 1465 (C–H), 1700, (C=C), and 1636 and 815 (C=C). ^1^H NMR (δ, DMSO-*d*_6_, 400 MHz, ppm): 6.05–6.12 (m, 2H,–CHC–(furan)), 6.07–6.05 and 5.69–6.67 (m, 2H,–H_2_C=C–(methacrylate)), 4.35–4.33 (m, 2H,–NCH_2_C−), 4.11–4.08 (t,–CCH_2_O–(HEMA)), 3.60–3.53 (m, 4H,–CH_2_−), 3.51 (s,–CH_2_–(PEG backbone)), 1.89–1,87 (s,–CH_3_ (HEMA)), 1.51–1.31 (m,–CH_2_–(HDI backbone)). SEC _(*THF*)_: *M*_n_ = 1700 g mol^–1^, *Đ* = 1.21.

### 2.3 FBMO Characterization

FTIR spectra were recorded on a PerkinElmer Spectrum Spotlight 300 and analyzed with OMNIC software. ^1^H NMR spectroscopy was recorded on a BrukerDPX-400 spectrometer operating at a frequency of 400 MHz for protons using DMSO-*d*_6_ as a solvent to prepare solutions of 10% w/v. The number of scans was 64, and the sweep width was 9.6 kHz. Size exclusion chromatography (SEC) measurements were performed on waters e2695 separations module, Waters Corporation, USA, using tetrahydrofuran as the injection eluent at 35 °C with the flow rate of 1 mL/min, equipped with PLgel 10 μm mixed B2 columns. All SEC samples were passed through 0.45 μm nylon-66 filter membranes before analysis. Narrow standard polystyrene was used for calibration between 1220 and 1.2 × 10^6^ Da and monitored by refractive index (RI) detection.

### 2.4 Photosensitive Resin Formulation

In the preparation of photosensitive resins, different compositions of FBMO and methacrylate-containing compounds (i.e., PEGDMA or TEGDMA) were first mixed (as shown in Table S1) and magnetically stirred for 60 min. In each photosensitive resin, Hydroquinone was added at 0.1 wt % of the mixture, followed by 2.9 wt % of TPO. The solution mixtures were continuously stirred overnight. The codes of formulated photosensitive resin were assigned based on the types and amount of FBMO and methacrylate-containing compounds used, for example, FBMO-50-PEG-50 formulated from FBMO (48.5 wt %) and PEGDMA (48.5 wt %). The rheological analyses were conducted using a rheometer equipped with a cone plate (the gap between the plates of 70 μm). All analyses were done within a shear rate range of 1–100 s^–1^ at room temperature. The curing depth of each formulated resin was obtained by following the protocol previously reported. Typically, a photosensitive resin was dropped in a circular shape with approximately 3 mm diameter at the center of the resin vat and exposed to near UV light for 40 s (405 nm, ELEGOO Saturn S resin 3D printer). Afterward, the unreacted photosensitive resin was carefully removed, and the thickness of the cured specimen (*n* = 5) was measured with a digital vernier caliper (Japan Mitutoyo 500–197–20/30200 mm/8).

### 2.5 Fabrication of the 3D-Printed Specimen

The 3D-printed specimens were fabricated by the ELEGOO Saturn S resin 3D printer using the designs (cylindrical and gyroid structures, Figure S1) prepared through computer-aided design (CAD) software, Autodesk Netfabb (Autodesk). In the design, the diameter and height of a CAD model were set at 8 and 3 mm, respectively. Three gyroid structures, with different wall thicknesses and average pore sizes ranging from 400 to 800 μm and 1.1–1.7 mm, respectively, were fabricated in this work. The printed layer thickness was set at 100 μm; each layer was exposed to near UV light (405 nm) for 15 s. After printing, the green specimens were cleaned in isopropanol for 2 min via ultrasonication and in deionized water for 2 min to remove any excess resin on the material surfaces. Afterward, the green specimens were exposed to UV light for 20 min (10 min for each side) using a UV lamp with a lamp power of 40 W and a light intensity of 3.40 mW/cm^2^ (noted as post-cured specimens). The codes of the 3D-printed specimen were assigned based on the design, wall thickness (porous structure), and formulation used; for example, CD-FBMO-90-PEG-10 fabricated from FBMO-90-PEG-10 resin using the cylindrical model and GR400-FBMO-90-TEG-10 represented the gyroid specimen with 400 μm of wall thickness fabricated from FBMO-90-PEG-10 resin.

### 2.6 Determination of Double-Bond Conversion

The double-bond conversion (DBC) was measured using attenuated total reflection (ATR) with an FTIR spectrometer modified from the previous reports.^30,31^ Notably, the photosensitive resins were primarily analyzed. After analyzing green specimens and post-cured specimens, two major characteristic absorbances were used for the determination of DBC: one at 810 cm^–1^ (baseline 780–828 cm^–1^) corresponding to the bending vibration of the *H–C*=C bond and the other at 1714 cm^–1^ (baseline 1678–1756 cm^–1^) corresponding to the stretching vibration of the C=O bond. At the time of photopolymerization, the peak area of the *C*=*C* bond observed in the cured specimen subsided, whereas the peak area of the *C*=*O* bond was unperturbed and consequently used as a reference to normalize the peak area value. The peak areas of both signals were calculated after baseline correction using OMNIC software. Subsequently, the double-bond conversion in the cured specimen was then calculated by eq 1 below:
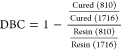
1where cured (810) and cured (1716) are peak areas of the C=C and C=O of the printed samples, respectively, resin (810) and resin (1716) represent the peak areas of the C=C and C=O of the photosensitive resins, respectively. Three repetitive measurements were collected for each sample to calculate the mean value and standard deviation.

### 2.7 Analysis of Mechanical and Thermal Properties

The mechanical property of the post-cured specimens was evaluated by a compressive test conducted at room temperature using a universal testing machine (UTM, mechanical analyzer (Texture Analyzer) (Shimadzu/EZ-Test (EZ-LX)) equipped with a load cell of 1 kN. The specimen (*n* = 3) was compressed with a 0.5 mm/min crosshead speed until mechanical failure. The compressive modulus was determined from the slope of the linear portion of the stress–strain curve. Thermogravimetric analysis (TGA) has been applied to investigate the thermal behavior of 3D-printed specimens. TGA instrument (Mettler Toledo, Hong Kong) with a constant heating rate of 10 °C/min was employed to characterize the post-cured specimens. Each sample was heated from ambient temperature to 600 °C under a 60 mL/min nitrogen flow purge.

### 2.8 Surface Morphology Assessment

The morphology of the post-cured specimens was observed by scanning electron microscopy (SEM, Hitachi S-3400N) with an applied voltage of 20 kV and a working distance of 10 mm. The surface of each specimen was sputter-coated with Au for 120 s at 15 mA to ensure conductivity. Surface morphology was observed from the top and cross-sectional views of the final printed layer. A specimen was cut vertically with a razor blade to conduct the cross-sectional area.

### 2.9 Cytotoxicity Evaluation

#### 2.9.1 Auricular Chondrocyte Isolation and Culture

Porcine auricular chondrocytes were isolated from the auricular cartilage tissues of born dead pigs (*Sus scrofa*). The auricular cartage specimens were rinsed in a culture medium containing 1% P–S. The samples were then aseptically cut into small pieces. They were enzymatically digested in a digestion medium consisting of DMEM, 0.3% collagenase I, and 1% P–S at 37 °C in a CO_2_ incubator overnight. The auricular chondrocytes were collected by trypsinization using 0.25% trypsin-EDTA and cultured in a culture medium (DMEM, 1% P–S, 10% FBS, and 1% NEAA) at 37 °C in the incubator supplied with 5% of CO_2_. The cells were passaged when they reached about 80% confluence. Cells were used in passage 3 for the cytotoxicity test.

#### 2.9.2 Cytotoxicity Test

Cytotoxicity of the 3D-printed scaffolds was examined using 3-[4,5-dimethylthiazol-2-yl]-2,5 diphenyl tetrazolium bromide (MTT) assay, which is based on the conversion of the water-soluble yellow MTT dye into the insoluble purple formazan crystals by living cells. Each scaffold extract was prepared according to the ISO 10993–5 procedure. In brief, the prepared scaffolds were washed using acetone/DI water (70/30%v/v) (3 × 40 mL) and subsequently sterilized using ethylene oxide before submerging them in a culture medium (0.1 g/mL) for 24 h at 37 °C in a 5% CO_2_ incubator. All extracts were sterilized by filtration using a 0.2 μm syringe filter. The primary auricular chondrocytes were plated at a density of 5 × 10^3^ cells per well in 96-well microplates in 200 μL of culture medium. After 48 h of incubation, the culture medium was removed, and cells were exposed to 200 μL of either GR400-FBMO-90-PEG-10 or GR400-FBMO-90-TEG-10 extract for 48 h. At the end of the treatment, the extract was replaced with 200 μL culture medium containing 0.5 mg/mL MTT and left to incubate for a further 4 h. Afterward, the medium containing MTT was discarded, and the formazan crystals were dissolved with 200 μL of dimethyl sulfoxide (DMSO; Corning) solution. The obtained lysate was measured at 570 nm using a VICTOR X4 multilabel plate reader (PerkinElmer). The results were reported as the percentage of viability relative to the untreated control (negative control was regarded as 100% of viability).

### 2.10 Statistical Analysis

Data are presented as mean ± standard deviation (SD). Statistical analyses were performed using SPSS program version 19.0 (SPSS, Inc., Chicago, IL) with a one-way ANOVA followed by Duncan’s multiple range test. The differences were considered significant and marked with different letters when *p* < 0.05.

## 3 Results and Discussion

### 3.1 FBMO Preparation

**Scheme 1:**
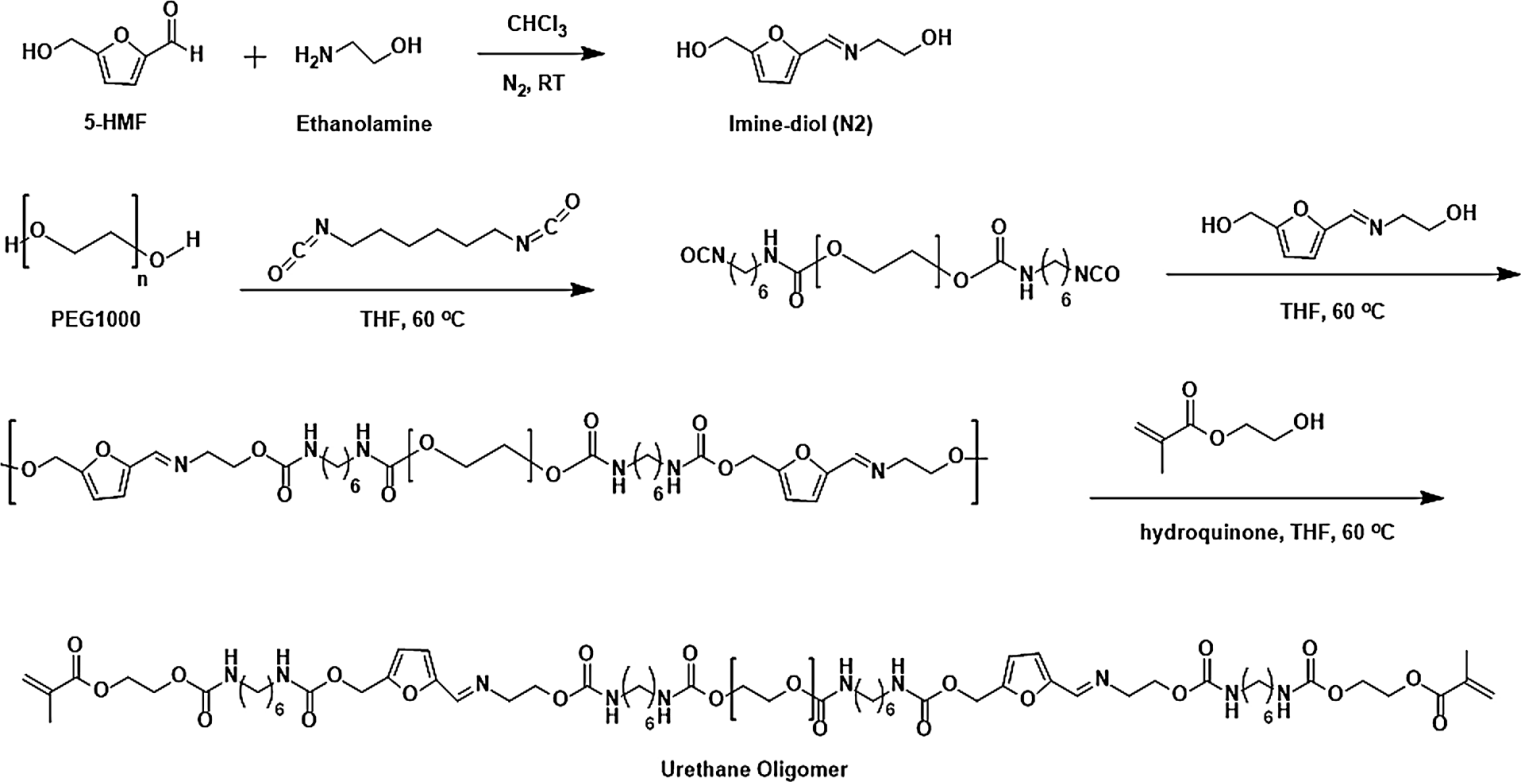
Synthesis of FBMO with the Molar Ratio of PEG1000, HDI, N2, and HEMA at 1:6:3:3, Respectively

The synthetic pathway for Monomer N2 and FBMO has been elucidated in Scheme 1. Initially, Monomer N2 was synthesized through an imination reaction involving 5-HMF and amino ethanol, forming a new furan-imine diol. Subsequently, FBMO was synthesized in two steps. In the first step, PEG-1000 and HDI were sequentially reacted with Monomer N2 to produce a preoligomer. Next, the preoligomer chains were end-capped with HEMA to yield the furan-based methacrylate oligomer. The molar ratio of PEG1000, HDI, N2, and HEMA was set at 1:6:3:3, respectively, to ensure the end-capping with HEMA at the HDI chain end in the final step. It is worth noting that the synthesized FBMO contains 23% biobased content, sourced from the quantity of 5-HMF.

**Figure 1:**
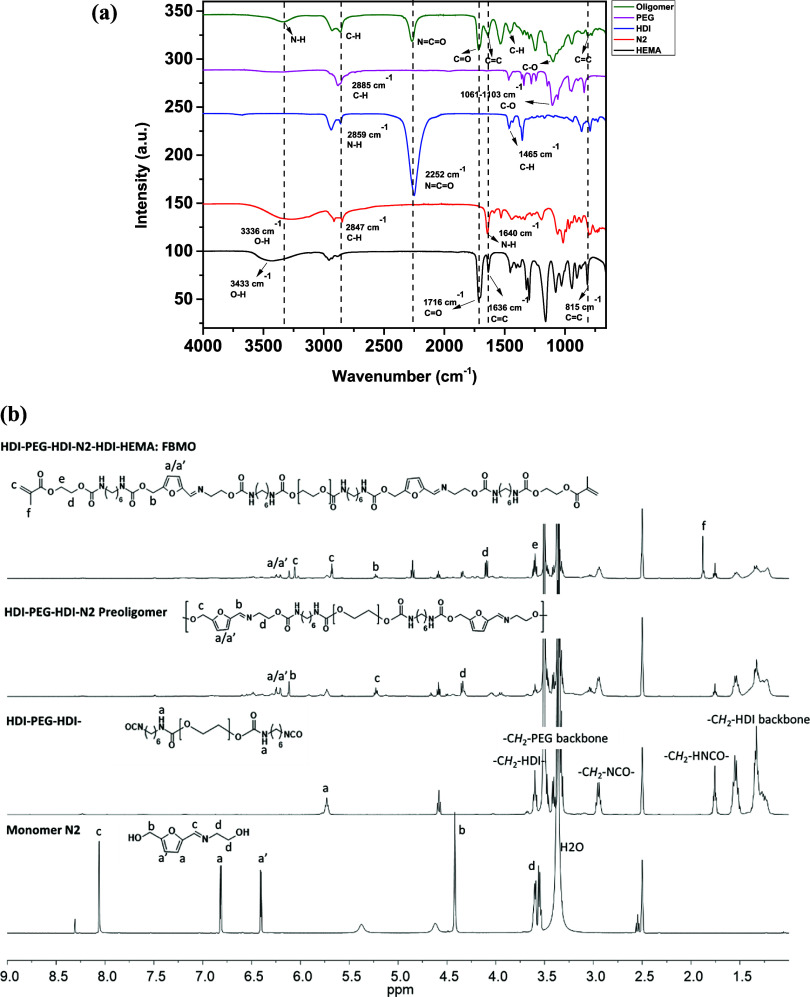
(a) ATR-FTIR spectra of HEMA, monomer N2, HDI, PEG1000, and oligomer (FBMO) and (b) stacked ^1^H NMR spectra of FBMO synthesis at each step.

The chemical structure of the resultant oligomer was confirmed through FTIR and ^1^H NMR measurements, and the FTIR spectra of all chemicals and FBMO as well as ^1^H NMR spectra in each synthesis step are illustrated in Figure 1. The FTIR spectrum exhibits characteristic bands of methacrylate oligourethane, including N–H stretching at 3336 cm^–1^, antisymmetric and symmetric C–H stretching of CH_2_ at 2885 and 2859 cm^–1^, C=O stretching of urethane at 1716 cm^–1^, C=C stretching of methacrylate at 1636 cm^–1^, C=C bending of methacrylate at 815 cm^–1^, C=N stretching at 1641 cm^–1^, N–H bending at 1594 cm^–1^, C–N stretching at 1020 cm^–1^, and CH_2_ bending at 1447 cm^–1^. However, the absorption peak of–NC=O groups from HDI at 2252 cm^–1^ persisted in FBMO, which did not impede the subsequent formulation of photosensitive resin. The analysis of the ^1^H NMR spectra of FBMO synthesis at each step revealed characteristic signals that are consistent with the corresponding structures. As observed in Figure 1b, the signal of isocyanate moieties is clearly visible prior to end-capping with HEMA in the final step. The ^1^H NMR spectrum of FBMO (Figure S3, 400 MHz, DMSO-*d*_6_) showed all assigned peaks, confirming the oligomer structure. The size exclusion chromatography (SEC) technique revealed a number-average molecular weight (*M*_n_) of 1700 g mol^–1^ with dispersities (*Đ*) of approximately 1.21, affirming the controlled character of the oligomer (Figure S4). Consequently, the combined results of FTIR and SEC analyses support the successful synthesis of FBMO.

### 3.2 Resin Formulation and Analysis

**Figure 2:**
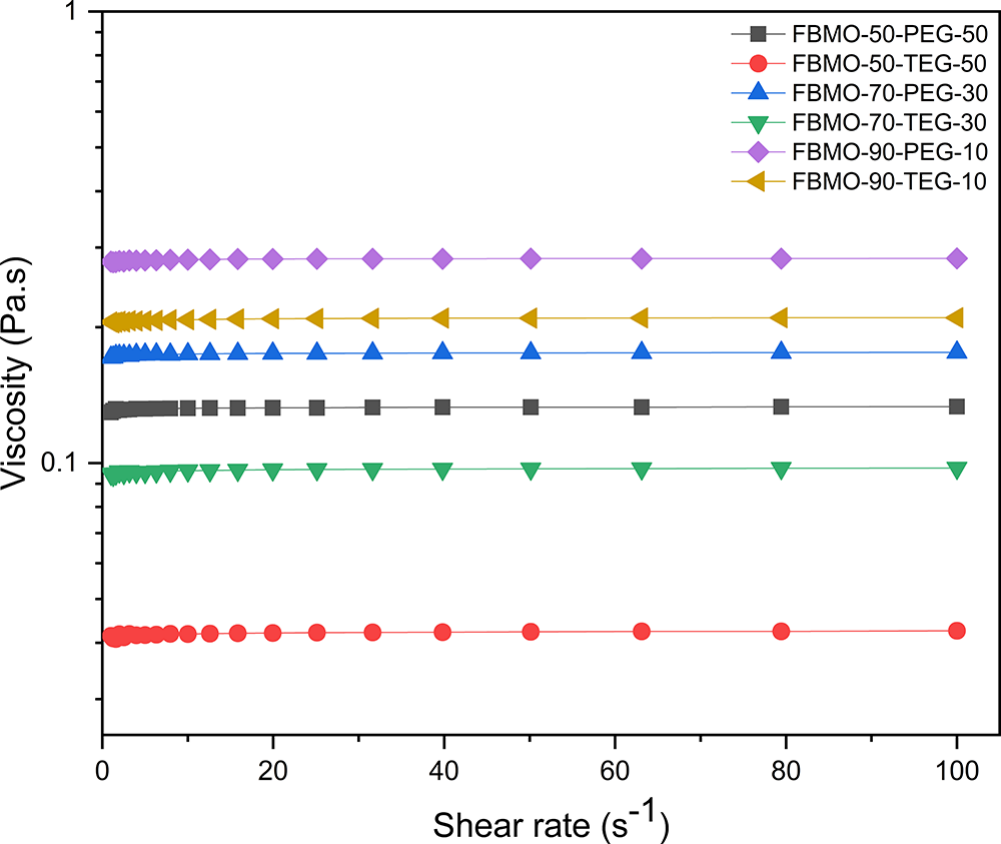
Viscosity of photosensitive resins as a function of the shear rate measured at 25 °C.

The photosensitive resins were formulated from different resin compositions of FBMO and methacrylate-containing compounds (i.e., PEGDMA or TEGDMA) to investigate their influences on resin characters and, indeed, the properties of 3D-printed specimens. The overall biobased content in FBMO-TEGMA or PEGMA can reach up to 20%. Notably, the purposes of incorporating methacrylate-containing compounds were to reduce the viscosity and synchronously increase the printing fidelity as the concentration of polymerizable groups of the photosensitive resins increased.^32^ The viscosities of all photosensitive resins formulated were measured by a cone rheometer within a shear rate range of 1–100 s^–1^. Regarding the data shown in Figure 2, all of the prepared resins in this study exhibited Newtonian behavior, and the viscosity fell in the range of 0.04 to 0.28 Pa s at a shear rate of 10 s^–1^, depending on the amount and type of methacrylate-containing compounds blended in the resins. Indeed, PEG-containing photosensitive resin yielded a higher viscosity than TEG-containing resin; likewise, a similar result was obtained when higher FMBO content was incorporated. This suggests that the greater viscosity of the resin formulated from the high molecular weight polymer was due to the enlargement of chain entanglements in the system.

Before the fabrication process, the study of photosensitive resin reactivity (also known as curing depth study) was determined to ensure the printability of the formulated resin. Each formulation was individually exposed to the 3D printer light source for 40 s. Subsequently, the cured samples with different thicknesses in the range of 0.44 to 0.64 μm were obtained, as depicted in Table S2. Apparently, the curing depths of FMBO-dominated photosensitive resin were significantly shallower (*p* < 0.05) than those of the resins with a high methacrylate-containing compound content, belonging to the less reactive groups in the formulated resins. This indicates that the amount of photopolymerizable groups directly influences resin reactivity. Notably, the thickness of the cured TEG-containing resins was somewhat different from that of the cured PEG-containing resins.

### 3.3 Fabrication of 3D-Printed Specimens

**Figure 3:**
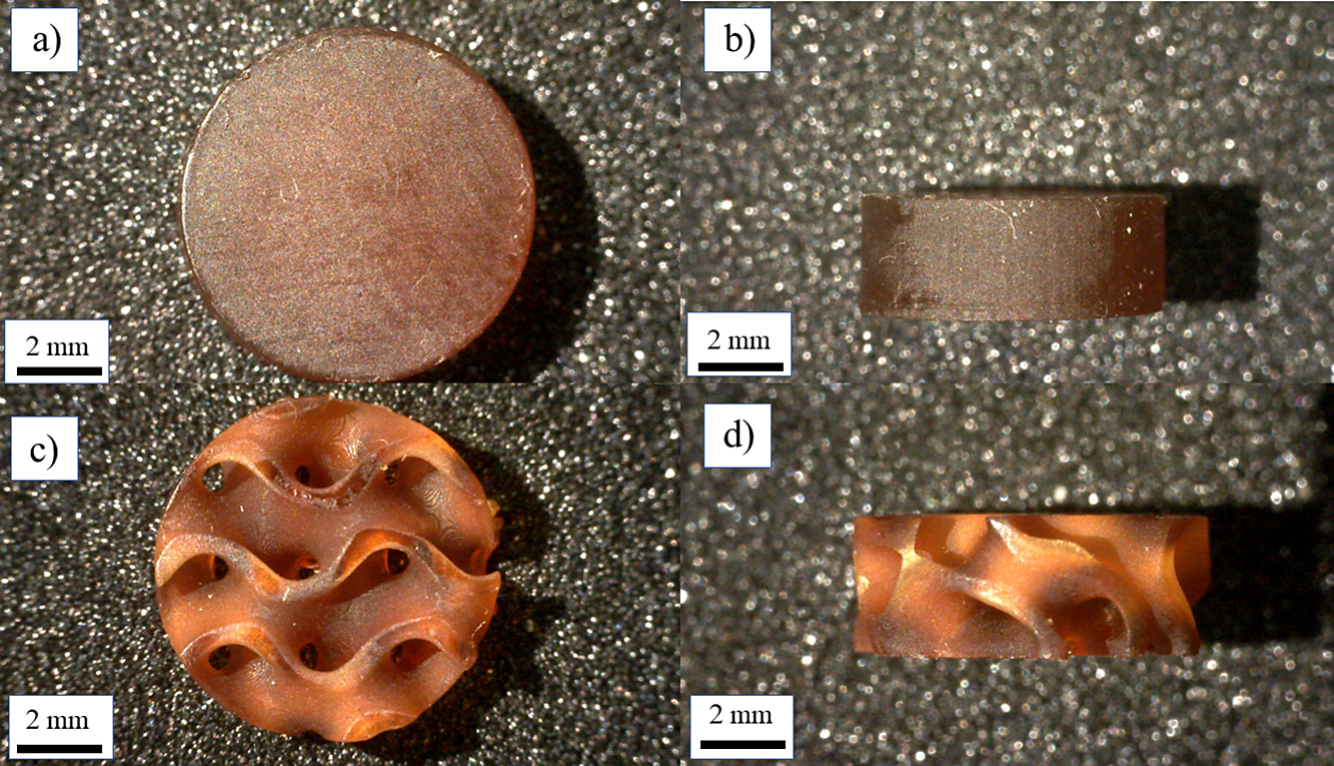
Aspect of the post-cured specimens fabricated from FBMO-90-TEG-10 resin, (a, b) cylindrical structure (CD-FBMO-90-TEG-10), and (c, d) gyroid structure (GR400-FBMO-90-TEG-10).

The printing fidelity of photosensitive resin composed of FBMO in this study was monitored with respect to the accuracy of the printed specimens using two different CAD models: cylindrical and gyroid structures. Notably, three gyroid structures were fabricated with varying thicknesses of wall and average pore sizes ranging from 400 to 800 μm and 1.1–1.7 mm, respectively. The 3D-printed specimens were fabricated by layer-by-layer photopolymerization with a layer thickness of 100 μm, which was relevant to curing depths of the printing resins mentioned above to achieve good adhesion between layers with the minimum of overphotopolymerization effect. Subsequently, the 3D-printed specimens were cleaned through ultrasonication and in isopropanol and deionized water before postcuring for 30 min (15 min for each side) using a UV lamp. The structure of all post-cured samples was apparently well in accordance with the CAD model, as shown in Figure 3; this observation indicates that the formulated photosensitive resin offers reasonable printing accuracy.

The size of post-cured specimens was determined using the digital vernier caliper, as displayed in Table S3; overall, it was seemingly related to the size CAD model but slightly different from the design, particularly, the diameter of the porous structure. Interestingly, the height of the gyroid samples was revealed to be marginally larger than the model by about 3%. This observation is likely attributed to overphotopolymerization in the *Z*-axis during printing. In addition, the slow photopolymerization rate, as the curing time for each layer is quite long (about 15 s per printing layer), may result in the inaccuracy of specimens prepared through the stereolithography technique. Besides, gravity can induce the resin to drip toward the resin tank before achieving a reasonable DBC to solidify utterly. A similar observation was noticed for the fabrication of cylindrical structures, with approximately 4% of the specimen height being bulkier than the design. However, the structure’s diameter revealed unpredictable results, as shrinkage and enlargement of specimens were observed in the cylindrical construct. Notably, gyroid structures exhibited a smaller size than the design because the size of the 3D-printed porous structure is likely to lessen compared to bulk specimens.

### 3.4 Assessment of Double-Bond Conversion

**Figure 4:**
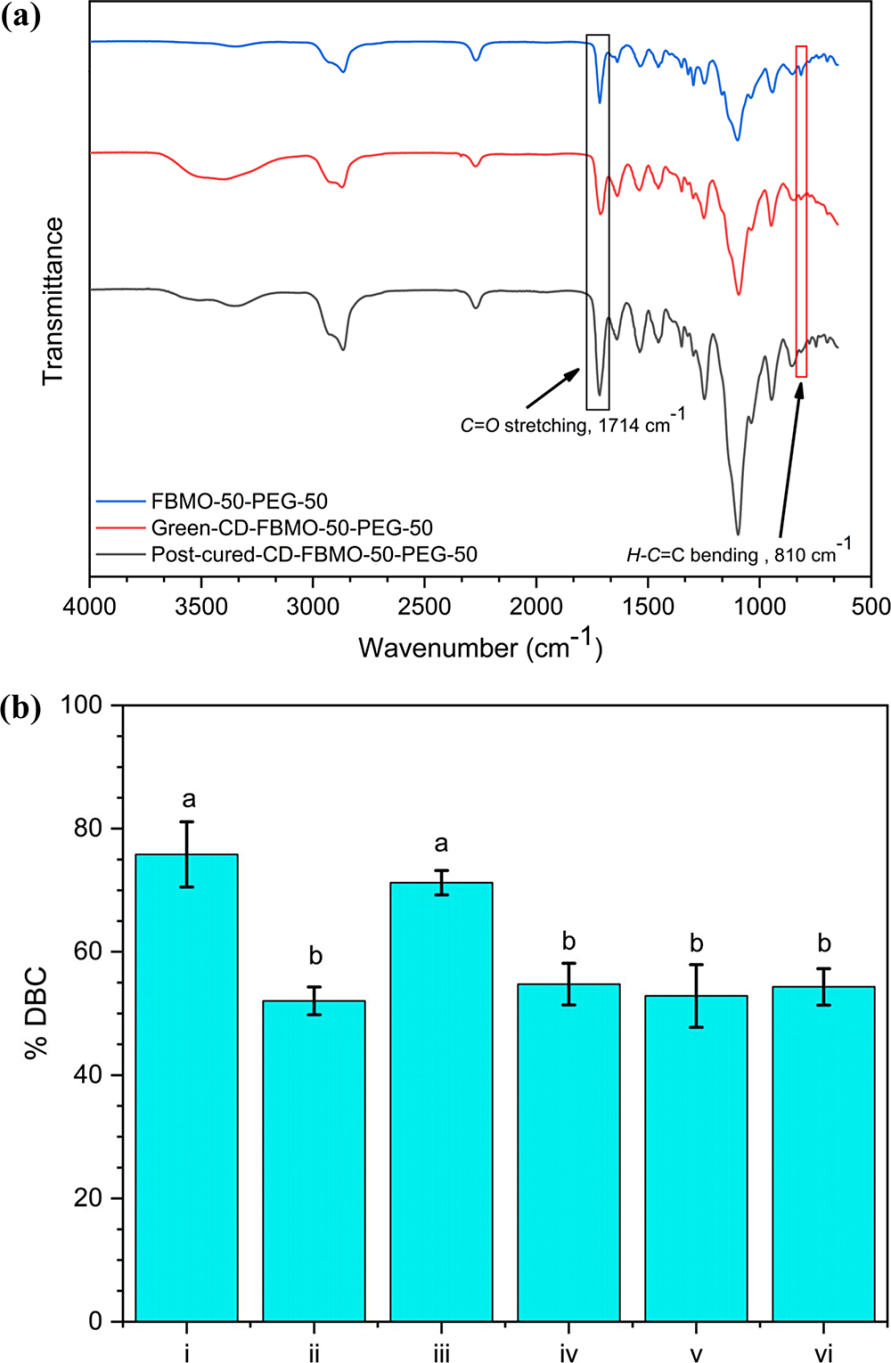
(a) ATR-FTIR spectra of photosensitive resin, printed green specimen, and post-cured specimen (CD-FBMO-50-PEG-50) and (b) DBC of post-cured specimens fabricated from different photosensitive resins ((i) CD-FBMO-50-PEG-50, (ii) CD-FBMO-50-TEG-50, (iii) CD-FBMO-70-PEG-30 (iv) CD-FBMO-70-TEG-30, (v) CD-FBMO-90-PEG-10, and (vi) CD-FBMO-90-TEG-10). Different lowercase letters indicate significant differences in the thickness of cured samples at *p* < 0.05.

Fabricating 3D-printed specimens through stereolithography is the solidification process of photosensitive resin. Indeed, studying the polymerizable monomer/polymer transformation in the resin is substantial. The main characteristic absorbances were used to determine DBC: one at 810 cm^–1^ corresponding to the bending vibration of the *H–C*=C bond; the other at 1716 cm^–1^ corresponding to the stretching vibration of the *C*=*O* bond, as the number of *C*=*C* bonds in the resin instantly reduced during photopolymerization, whereas the number of *C*=*O* bonds was simultaneously unaltered, as shown in Figure 4a. The DBC in each specimen (cylindrical structure) was directly determined by eq 1, previously stated in Section 2.5. Figure 4b tabulates the double-bond conversion of printed green specimens and their corresponding post-cured specimens; DBCs found in the printed green specimens were in the range of 30–50%. Upon postcuring, the molecules of the uncured photosensitive in the green specimens became further photopolymerization caused by newly generated radicals induced by the UV light; thereby, the increased monomer/polymer conversion up to about 60–75% was achieved. Notably, postcuring is necessary to enhance the conversion of reactive groups in the specimens after printing.

### 3.5 Mechanical Properties of 3D-Printed Specimens

**Figure 5:**
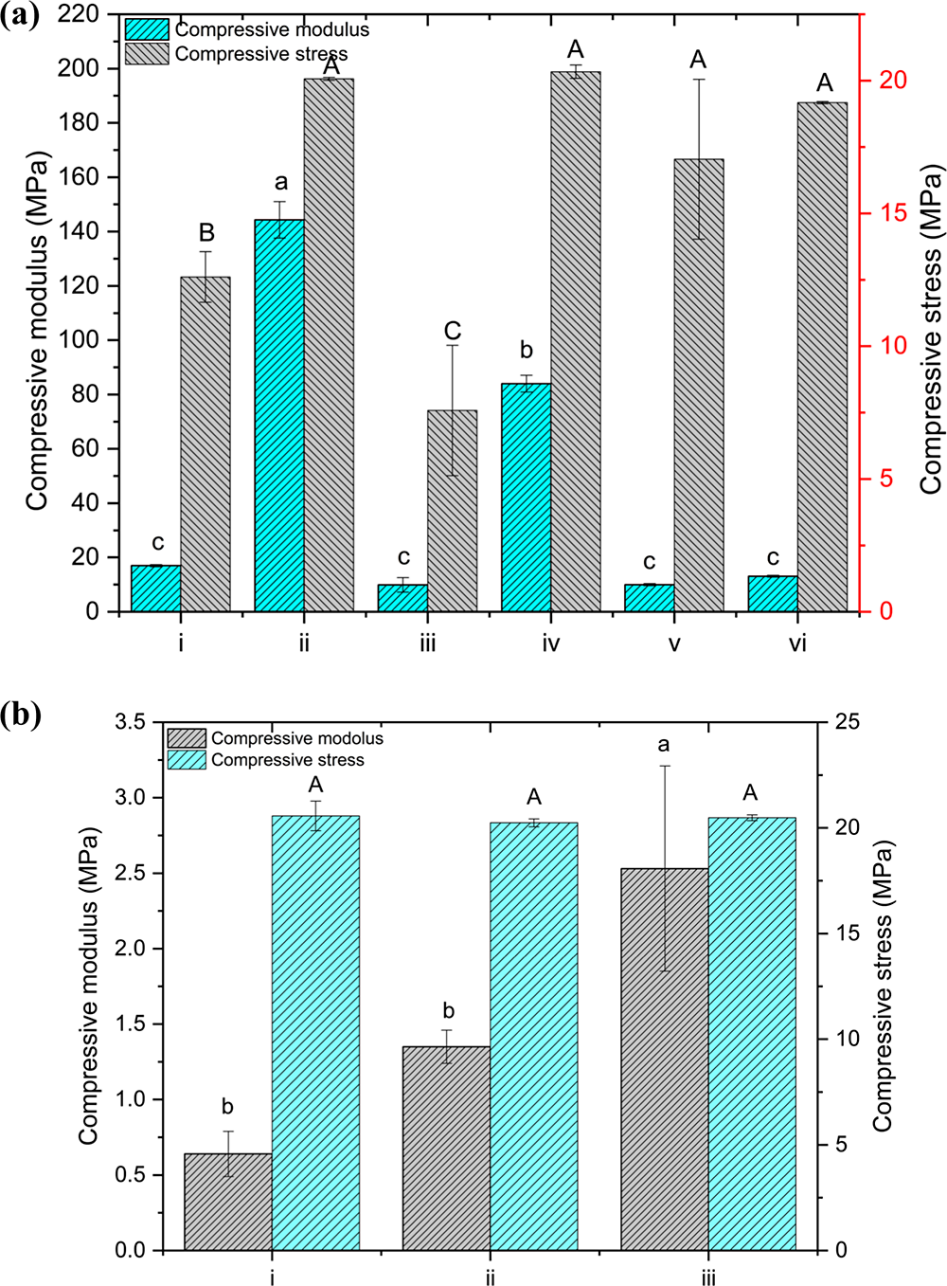
(a) Compressive modulus and compressive stress of all cylindrical specimens; (i) CD-FBMO-50-PEG-50, (ii) CD-FBMO-50-TEG-50, (iii) CD-FBMO-70-PEG-30 (iv) CD-FBMO-70-TEG-30, (v) CD-FBMO-90-PEG-10, and (vi) CD-FBMO-90-TEG-10), and (b) compressive modulus and compressive stress of gyroid structures; (i) GR400- FBMO-90-PEG-10, (ii) GR600- FBMO-90-PEG-10, and (iii) GR800- FBMO-90-PEG-10. Different lowercase letters indicate significant differences among the mechanical properties of the 3D-printed construct at *p* < 0.05.

Polyurethanes continue to gain interest in biomedical fields due to their unique flexibility. The mechanical property, one of the crucial features required for elastic material for biomedical applications, of various 3D-printed specimens was evaluated regarding compressive modulus. Initially, the cylindrical 3D-printed specimens fabricated from different compositions of FBMO and methacrylate-containing compounds were compressed using a universal testing machine equipped with a load cell of 1 kN to explore the resin formulation that could provide a flexible character. The compressive modulus and stress of all cylindrical specimens are represented in Figure 5a, with the corresponding stress–strain curve displayed in Figure S5a. They reveal that the compositions of FBMO and methacrylate-containing compounds greatly influence their mechanical behavior. Among these specimens, FBMO-dominated samples, CD-FBMO-90-TEG-10 and CD-FBMO-90-PEG-10, highlighted the most elastic behavior regarding the stress–strain relationship with a compressive modulus of 13.07 ± 0.35 and 9.98 ± 0.42 MPa, respectively. On the other hand, the stress–strain curve of CD-FBMO-50-TEG-50 and CD-FBMO-70-TEG-30 samples reveal brittle behavior, indicating that TEGDMA significantly influences the physical properties of the printed specimens over the FBMO in those formulations. Notably, the specimens prepared from PEGDMA-containing photosensitive resins exhibited a more elastic character. This observation was attributed to the longer chain length of PEGDMA compared to TEGDMA’s; a similar observation was reported as a lower molecular weight of polymer offered a stiffer 3D-printed specimen. Unfortunately, CD-FBMO-50-PEG-50 and CD-FBMO-70-PEG-30 exhibited mechanical failure after being compressed at 40 and 45% strain, respectively. This vividly suggested that the good flexibility of 3D-printed samples is associated with the flexible segment at the molecular level of FBMO and, conceivably, PEGDMA. Indeed, the FBMO-90-TEG-10 and FBMO-90-PEG-10 formulations were thus selected for further fabrication of the different cellular structure scaffolds.

Apart from adjusting resin formulation to achieve the specimens with the desired properties, tuning their cellular structure can also be considered an effective strategy. In this current study, gyroid structures, a fashionable triply periodic minimal surface (TPMS) that has been widely used in biomedical fields, particularly for elastic implant scaffolds owing to its ability to absorb compressive energy, were prepared using FBMO-90-TEG-10 and FBMO-90-PEG-10 formulations. Three gyroid structures, with different wall thicknesses and average pore sizes ranging from 400 to 800 μm and 1.1–1.7 mm, respectively, were fabricated to study the effect of internal architecture on their mechanical performance. The resulting gyroid-printed specimens fabricated using the layer thickness and exposure time of 100 μm and 12 s exhibited elastic performance, as the compressive modulus and stress depicted in Figure 5b, with the corresponding stress–strain curve displayed in Figure S5b. The compressive modulus of the porous specimen was in the range of 0.53 ± 0.04 to 4.50 ± 0.40 MPa, depending on the wall thickness and methacrylate-containing compound in the formulation. Expectedly, the mechanical behavior of the gyroid structures is directly proportional to their wall thickness, for example, the compressive modulus of GR800-FBMO-90-PEG-10 is higher than GR200-FBMO-90-PEG-10 by approximately 5 times. A similar tendency of the gyroid structure prepared from FBMO-90-TEG-10 formulation was noticed. Besides, the compressive modulus of GR800-FBMO-90-TEG-10 was significantly (*p* < 0.05) greater than GR800-FBMO-90-PEG-10 about 1.8 times. This was likely associated with a higher polymerizable group per mole of TEGDMA than PEGDMA, leading to a greater cross-linking density. Besides, the thermal behaviors of the printed specimens were examined by TGA; indeed, a similar technique has been reported for highlighting the thermal stability of 3D printed structures.^33−35^ Notably, two thermal characters corresponded to the evaporation of volatile unpolymerized resins and cross-linked resin after polymerization. The evaporation of volatile events was noticed at about 130 °C for all samples (Figure S6). Besides, the cross-linked region started decomposing at approximately 250 °C and nearly completely burnt out at 450 °C. We detected a 50% weight loss of the post-cured specimens, that is, FBMO-50-TEG-50, FBMO-70-TEG-30, FBMO-90-TEG-10, FBMO-50-PEG-50, FBMO-70-PEG-30, and FBMO-90-PEG-10, achieved after the temperature reached 393, 411, 412, 405, 411, and 408 °C; this revealed that the thermal stability of the fabricated specimen herein is relatively tolerable. Overall, these results showed that the bulk and porous specimens prepared from FBMO in this study offered unique flexibility and thermal stability. In addition, the fabrication of specimens using the cellular structure also opens up opportunities to alter and improve the mechanical properties to obtain materials with a promising elastic recovery behavior, which can be further utilized for appropriate biomedical and other applications.

### 3.6 Morphological Properties of 3D-Printed Specimens

**Figure 6:**
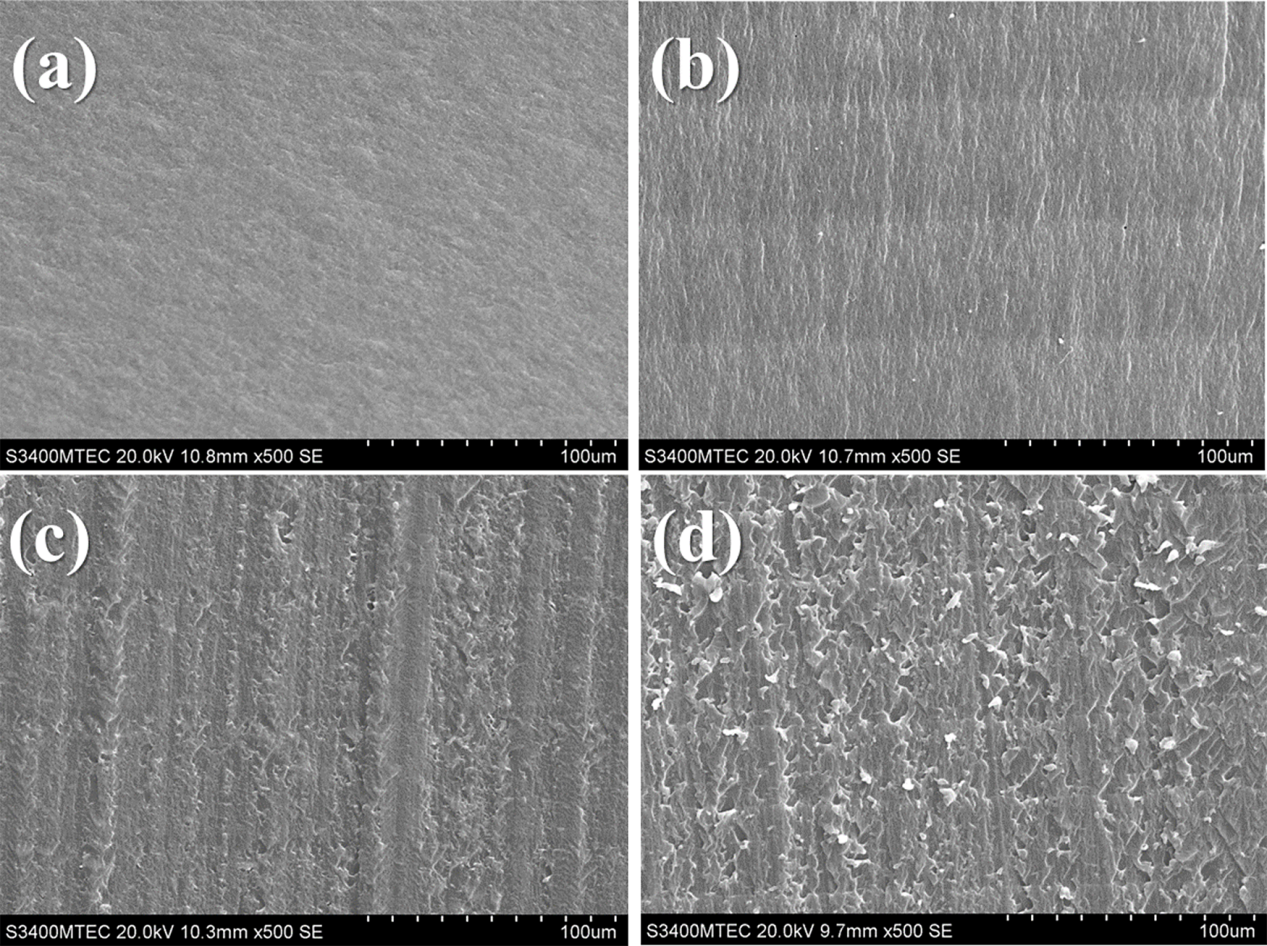
SEM micrographs of post-cured specimens (cross-sectional area): (a) CD-FBMO-50-TEG-50, (b) CD-FBMO-70-TEG-30, (c) CD-FBMO-90-TEG-10, and (d) CD-FBMO-90-TEG-10.

SEM micrographs were taken from cross-sectional views of selected post-cured specimens to investigate the influences of methacrylate-containing compounds on the surface morphology of 3D-printed specimens. Figure 6 exhibits the SEM images of post-cured specimens (cross-sectional area, 500×) prepared from different formulations: FBMO-50-TEG-50, FBMO-70-TEG-30, FBMO-90-TEG-10, and FBMO-90-PEG-10. The SEM results indicate that the methacrylate-containing compound content considerably affects the morphological properties. The surface pixel of high methacrylate-containing compound content, CD-FBMO-50-TEG-50, was sharper than those of high FBMO content, CD-FBMO-90-TEG-10. Indeed, the surface of CD-FBMO-90-PEG-10 displayed some cured resin debris. This observation was likely associated with the longer polymer chain of PEGDMA compared to TEGDMA and, subsequently, the slower polymerization rate, leaving some partially cured resin on each printed layer.

Besides, the interconnectivity of porous specimens was affirmed through optical and SEM images of the 3D-printed construct, as displayed in Figure 3. The morphological study revealed reasonable printing accuracy of the gyroid specimens fabricated from FBMO-dominated formulations, FBMO-90-TEG-10 and FBMO-90-PEG-10, and a well-defined structure. The average wall thickness and pore size diameter of the posted-cured gyroid specimens directly measured from their SEM micrographs using ImageJ software were 600–900 μm and 1.0–1.6 mm, respectively (Figure S7). Interestingly, the wall thickness obtained at the top view of all gyroid specimens was larger than the design by approximately 30%, and the pore size was smaller than the model by about 10%, in particular, when considering the information obtained from the cross-sectional views. This observation was likely attributed to the low reactivity of formulated resins, as mentioned in the curing depth study section. Consequently, delayed photopolymerization during the printing process may cause the accumulation of the photosensitive resin between layers, leading to a marginally bulkier wall thickness than the CAD model. Overall, we have introduced the approach to prepare the photosensitive resin mainly composed of FBMO and fabricated porous structures (e.g., gyroid) with adjustable mechanical properties using light-assisted 3D printing.

### 3.7 Cytotoxicity Evaluation

**Figure 7:**
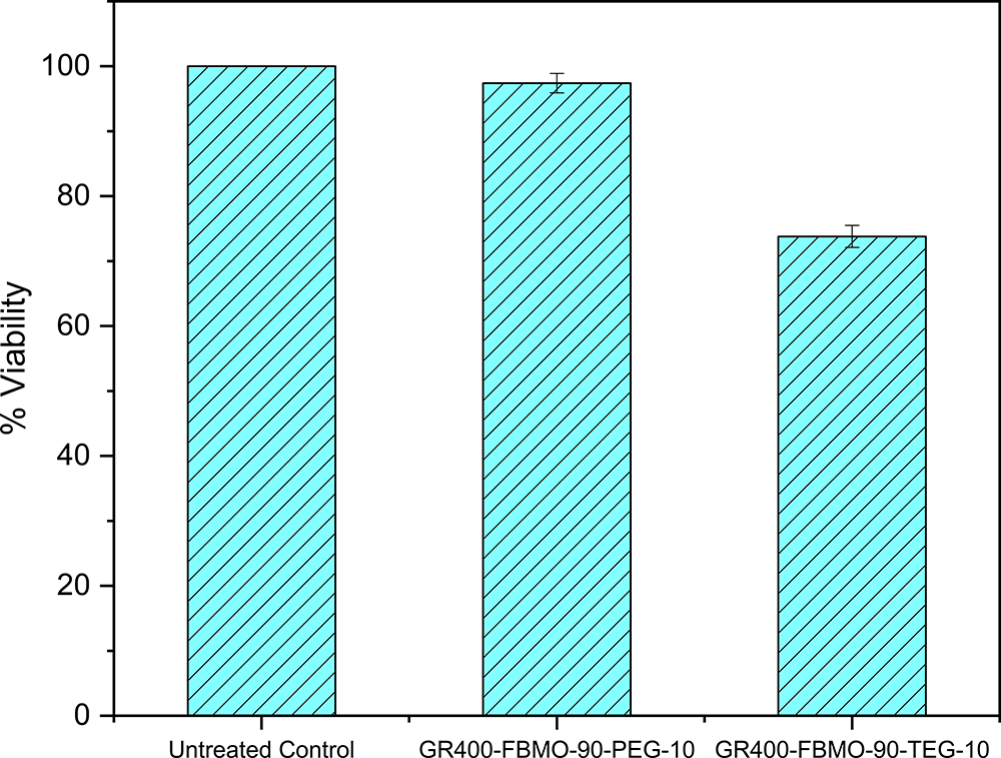
Cytotoxicity was tested by MTT assay on the porcine auricular chondrocytes after 48 h of incubation with liquid extracts of GR400-FBMO-90-PEG-10 and GR400-FBMO-90-TEG-10 specimens. Data were presented as the mean of % cell viability compared to the untreated control (*n* = 3).

As shown in Figure 7, the auricular chondrocytes exposed to the GR400-FBMO-90-PEG-10 and GR400-FBMO-90-TEG-10 extracts for 48 h revealed the percentages of cell viability at 97.4 ± 1.5 and 73.8 ± 1.7% of the untreated control, respectively. Notably, the gyroid structure with the highest content of FBMO was selected to test the cytotoxicity in this study because the gyroid structure has a higher surface area to contact with cells compared to the cylindrical structure, and we speculate that if the highest content of FBMO is nontoxic, indeed, less content should exhibit the similar result. The samples used in cytotoxicity evaluation were further purified by washing with acetone/DI water (70/30%v/v) before being sterilized using ethylene oxide. FTIR spectrum displayed the absence of an isocyanate (NCO) signal, which may cause cell toxicity at about 2250 cm^–1^, as depicted in Figure S8. The viability of cells in contact with the extracts was higher than 70%; thus, the result affirmed that the concentration of the substances released from the prepared scaffolds into the culture medium was lower than the toxic dose. According to the ISO EN 10993–5 criteria, these two scaffolds have cytocompatibility with the auricular chondrocytes.

## 4 Conclusions

The synthesis of FBMO containing imine functional groups has been successfully achieved through an imination reaction, followed by oligomerization. Subsequently, the FBMO with biobased content of 23% was employed in formulating photosensitive resin in combination with methacrylate-containing diluents. Rheological and cure-depth studies have unveiled that the resin’s rheological behavior and reactivity are contingent on the type and quantity of methacrylate-containing compounds. Smaller molecules, such as TEGDMA, exhibited lower viscosity but higher resin reactivity. The 3D-printed specimens demonstrated close adherence to the computer-aided design (CAD) models. Notably, the height of the cylindrical construct was found to be larger than the design by approximately 4%, likely attributed to the slow photopolymerization rate and the influence of gravity. As anticipated, the gyroid structures exhibited a smaller size compared to the design potentially due to a reduction in the porous structure compared to bulk specimens. Indeed, the physical properties of the 3D-printed specimens, including printing fidelity, mechanical properties, and morphology, could be finely tuned by controlling the ratios of FBMO to methacrylate-containing diluents and adjusting their internal structure. Cytotoxicity assessment results indicated that the 3D-printed specimens were noncytotoxic to porcine chondrocytes. In conclusion, we introduce a new strategy to prepare the furan-based methacrylate oligomer (FBMO) and 3D-printed specimens with adjustable properties using stereolithography, which can be further utilized for appropriate applications.

## Supporting Information Available

CAD models designed using Autodesk Netfabb software; characterization of Monomer N2 and FBMO by ^1^H NMR and SEC analysis; the size of 3D-printed specimens fabricated from different resin formulations using 3D printer and their analysis; stress–strain curve; thermal stability; surface morphology by SEM, and FTIR characterization (PDF)

## References

[ref1] Fei X.; Wang J.; Zhang X.; Jia Z.; Jiang Y.; Liu X. Recent Progress on Bio-Based Polyesters Derived from 2,5-Furandicarbonxylic Acid (FDCA). Polymers 2022, 14, 625 10.3390/polym14030625. 35160613 PMC8838965

[ref2] Zhu J.; Cai J.; Xie W.; Chen P. H.; Gazzano M.; Scandola M.; Gross R. A. Poly(Butylene 2,5-Furan Dicarboxylate), a biobased Alternative to PBT: Synthesis, Physical Properties, and Crystal Structure. Macromolecules 2013, 46 (3), 796–804. 10.1021/ma3023298.

[ref3] Papageorgiou G. Z.; Papageorgiou D. G.; Tsanaktsis V.; Bikiaris D. N. Synthesis of the Bio-Based Polyester Poly(Propylene 2,5-Furan Dicarboxylate). Comparison of Thermal Behavior and Solid State Structure with Its Terephthalate and Naphthalate Homologues. Polymer (Guildf) 2015, 62, 28–38. 10.1016/j.polymer.2015.01.080.

[ref4] Wang J.; Liu X.; Jia Z.; Sun L.; Zhu J. Highly Crystalline Polyesters Synthesized from Furandicarboxylic Acid (FDCA): Potential Bio-Based Engineering Plastic. Eur. Polym. J. 2018, 109, 379–390. 10.1016/j.eurpolymj.2018.10.014.

[ref5] Sheng Y.; Tan X.; Zhou X.; Xu Y. Bioconversion of 5-Hydroxymethylfurfural (HMF) to 2,5-Furandicarboxylic Acid (FDCA) by a Native Obligate Aerobic Bacterium, Acinetobacter Calcoaceticus NL14. Appl. Biochem. Biotechnol. 2020, 192 (2), 455–465. 10.1007/s12010-020-03325-7. 32394319

[ref6] Liu S.; Cheng X.; Sun S.; Chen Y.; Bian B.; Liu Y.; Tong L.; Yu H.; Ni Y.; Yu S. High-Yield and High-Efficiency Conversion of HMF to levulinic Acid in a Green and Facile Catalytic Process by a Dual-Function Bro̷nsted-Lewis Acid HScCl4Catalyst. ACS Omega 2021, 6 (24), 15940–15947. 10.1021/acsomega.1c01607. 34179638 PMC8223403

[ref7] Xia H.; Xu S.; Hu H.; An J.; Li C. Efficient Conversion of 5-Hydroxymethylfurfural to High-Value Chemicals by Chemo- and Bio-Catalysis. RSC Adv. 2018, 8, 30875–30886. 10.1039/C8RA05308A. 35548764 PMC9085621

[ref8] Xiang Y.; Wen S.; Tian Y.; Zhao K.; Guo D.; Cheng F.; Xu Q.; Liu X.; Yin D. Efficient Synthesis of 5-Ethoxymethylfurfural from Biomass-Derived 5-Hydroxymethylfurfural over Sulfonated Organic Polymer Catalyst. RSC Adv. 2021, 11 (6), 3585–3595. 10.1039/D0RA10307A. 35747695 PMC9134029

[ref9] Esen M.; Akmaz S.; Koç S. N.; Gürkaynak M. A. The Hydrogenation of 5-Hydroxymethylfurfural (HMF) to 2,5-Dimethylfuran (DMF) with Sol–Gel Ru-Co/SiO2 Catalyst. J. Solgel Sci. Technol. 2019, 91 (3), 664–672. 10.1007/s10971-019-05047-7.

[ref10] Post C.; Maniar D.; Voet V. S. D.; Folkersma R.; Loos K. biobased 2,5-Bis(Hydroxymethyl)Furan as a Versatile Building Block for Sustainable Polymeric Materials. ACS Omega 2023, 8, 8991–9003. 10.1021/acsomega.2c07629. 36936293 PMC10018510

[ref11] Moses V.; Narula A.; Chetan N.; Kumar Mishra R. Hydroxymethyl Furfural (HMF) a High Strength Cellulose Resin for Wood Composite Laminates. Heliyon 2022, 8 (12), e12081 10.1016/j.Heliyon.2022.e12081. 36544844 PMC9761700

[ref12] Chang H.; Motagamwala A. H.; Huber G. W.; Dumesic J. A. Synthesis of Biomass-Derived Feedstocks for the Polymers and Fuels Industries from 5-(Hydroxymethyl)Furfural (HMF) and Acetone. Green Chem. 2019, 21 (20), 5532–5540. 10.1039/C9GC01859J.

[ref13] Sailer-Kronlachner W.; Thoma C.; Böhmdorfer S.; Bacher M.; Konnerth J.; Rosenau T.; Potthast A.; Solt P.; Van Herwijnen H. W. G. Sulfuric Acid-Catalyzed Dehydratization of Carbohydrates for the Production of Adhesive Precursors. ACS Omega 2021, 6, 16641 10.1021/acsomega.1c02075. 34235336 PMC8246703

[ref14] Hevus I.; Kannaboina P.; Qian Y.; Wu J.; Johnson M.; Gibbon L. R.; Scala J. J. L.; Ulven C.; Sibi M. P.; Webster D. C. Furanic (Meth)acrylate Monomers as Sustainable Reactive Diluents for stereolithography. ACS Appl. Polym. Mater. 2023, 5 (11), 9659–9670. 10.1021/acsapm.3c02207.

[ref15] Papadopoulos L.; Malitowski N. M.; Bikiaris D.; Robert T. Bio-Based Additive Manufacturing Materials: An in-Depth Structure-Property Relationship Study of UV-Curing Polyesters from itaconic Acid. Eur. Polym. J. 2023, 186, 111872 10.1016/j.eurpolymj.2023.111872.

[ref16] Li X. J.; Wen Y. F.; Wang Y.; Zhou X. P.; Xie X. L. Modifying Poly(Propylene Carbonate) with Furan-Based Non-Isocyanate Polyurethanes. Chinese Journal of Polymer Science (English Edition) 2023, 41 (7), 1069–1077. 10.1007/s10118-023-2904-8.

[ref17] Olivito F.; Jagdale P.; Oza G. Synthesis and Biodegradation Test of a New polyether Polyurethane Foam Produced from PEG 400, L-Lysine Ethyl Ester Diisocyanate (L-LDI) and Bis-Hydroxymethyl Furan (BHMF). Toxics 2023, 11 (8), 698 10.3390/toxics11080698. 37624203 PMC10457969

[ref18] Huang Q.; Yang F.; Cao X.; Hu Z.; Cheng C. Thermally healable Polyurethanes Based on Furfural-Derived Monomers via Baylis-Hillman Reaction. Macromol. Res. 2019, 27 (9), 895–904. 10.1007/s13233-019-7123-3.

[ref19] Ngo T. D.; Kashani A.; Imbalzano G.; Nguyen K. T. Q.; Hui D. Additive Manufacturing (3D Printing): A Review of Materials, Methods Applications and Challenges. Composites Part B: Engineering 2018, 143, 172–196. 10.1016/j.compositesb.2018.02.012.

[ref20] Zhang H.; Lu D.; Yang S.; He Y.; Zhang H.; Bao J. A photocurable Polyurethane Elastomer for Digital Light Processing with Comprehensive Reusability Based on Dynamic Hindered Urea Bonds. Polymer (Guildf) 2024, 292, 126657 10.1016/j.polymer.2023.126657.

[ref21] Gama N.; Ferreira A.; Barros-Timmons A. 3D Printed Cork/Polyurethane Composite Foams. Mater. Des 2019, 179, 107905 10.1016/j.matdes.2019.107905.

[ref22] Samir A.; Ashour F. H.; Hakim A. A. A.; Bassyouni M. Recent Advances in Biodegradable Polymers for Sustainable Applications. npj Mater. Degrad. 2022, 6, 68 10.1038/s41529-022-00277-7.

[ref23] Chaudhary R.; Fabbri P.; Leoni E.; Mazzanti F.; Akbari R.; Antonini C. Additive Manufacturing by Digital Light Processing: A Review. Progress in Additive Manufacturing 2023, 8, 331–351. 10.1007/s40964-022-00336-0.

[ref24] Bagheri A.; Jin J. photopolymerization in 3D Printing. ACS Applied Polymer Materials 2019, 1, 593–611. 10.1021/acsapm.8b00165.

[ref25] Zhu G.; Zhang J.; Huang J.; Qiu Y.; Liu M.; Yu J.; Liu C.; Shang Q.; Hu Y.; Hu L.; Zhou Y. Recyclable and Reprintable biobased photopolymers for Digital Light Processing 3D Printing. Chemical Engineering Journal 2023, 452, 139401 10.1016/j.cej.2022.139401.

[ref26] Islam M. N.; Jiang Y. 3D Printable Sustainable Composites with Thermally Tunable Properties Entirely from Corn-Based Products. ACS Sustain Chem. Eng. 2022, 10 (24), 7818–7824. 10.1021/acssuschemeng.2c01806.

[ref27] Vyas J.; Shah I.; Singh S.; Prajapati B. G. Biomaterials-Based Additive Manufacturing for Customized Bioengineering in Management of Otolaryngology: A Comprehensive Review. Front. Bioeng. Biotechnol. 2023, 11, 1234340 10.3389/fbioe.2023.1234340. 37744247 PMC10515088

[ref28] Stouten J.; Schnelting G. H. M.; Hul J.; Sijstermans N.; Janssen K.; Darikwa T.; Ye C.; Loos K.; Voet V. S. D.; Bernaerts K. V. biobased photopolymer Resin for 3D Printing Containing Dynamic Imine Bonds for Fast Reprocessability. ACS Appl. Mater. Interfaces 2023, 15 (22), 27110–27119. 10.1021/acsami.3c01669. 37220092 PMC10251348

[ref29] Hu Y.; Zhu G.; Zhang J.; Huang J.; Yu X.; Shang Q.; An R.; Liu C.; Hu L.; Zhou Y. Rubber Seed Oil-Based Uv-Curable Polyurethane acrylate Resins for Digital Light Processing (Dlp) 3d Printing. Molecules 2021, 26 (18), 5455 10.3390/molecules26185455. 34576926 PMC8469773

[ref30] Rosa R. P.; Rosace G.; Arrigo R.; Malucelli G. Preparation and Characterization of 3D-Printed biobased Composites Containing Micro-or Nanocrystalline Cellulose. Polymers (Basel) 2022, 14 (9), 1886 10.3390/polym14091886. 35567055 PMC9105471

[ref31] Steyrer B.; Busetti B.; Harakály G.; Liska R.; Stampfl J. Hot Lithography vs. Room Temperature DLP 3D-Printing of a dimethacrylate. Addit Manuf 2018, 21, 209–214. 10.1016/j.addma.2018.03.013.

[ref32] Risangud N.; Jiraborvornpongsa N.; Pasee S.; Kaewkong P.; Kunkit N.; Sungkhaphan P.; Janvikul W. Poly(Ester-Co-Glycidyl Methacrylate) for Digital Light Processing in Biomedical Applications. J. Appl. Polym. Sci. 2021, 138 (42), 51391 10.1002/app.51391.

[ref33] Yao J.; Hakkarainen M. Methacrylated Wood Flour-Reinforced “All-Wood” Derived Resin for Digital Light Processing (DLP) 3D Printing. Compos. Commun. 2023, 38, 101506 10.1016/j.coco.2023.101506.

[ref34] Cosola A.; Sangermano M.; Terenziani D.; Conti R.; Messori M.; Grützmacher H.; Pirri C. F.; Chiappone A. DLP 3D – Printing of Shape Memory Polymers Stabilized by Thermoreversible Hydrogen Bonding Interactions. Appl. Mater. Today 2021, 23, 101060 10.1016/j.apmt.2021.101060.

[ref35] Abu Bakar A. A.; Zainuddin M. Z.; Abdullah S. M.; Tamchek N.; Mohd Noor I. S.; Alauddin M. S.; Alforidi A.; Mohd Ghazali M. I. The 3D printability and Mechanical Properties of Polyhydroxybutyrate (PHB) as Additives in Urethane dimethacrylate (UDMA) Blends Polymer for Medical Application. Polymers (Basel) 2022, 14 (21), 4518 10.3390/polym14214518. 36365512 PMC9657082

