# Supplementary material for: Synthesis and Characterization of Furan-Based Methacrylate Oligomers Containing the Imine Functional Group for Stereolithography
Source: ACS Omega. 2024 Jul 4;9(28):30771–81. doi: 10.1021/acsomega.4c03274 (PMC11256344; doi:10.1021/acsomega.4c03274)
Supplement: Supplementary file 1 — ao4c03274_si_001.pdf [file ao4c03274_si_001.pdf]

Synthesis and characterization of furan-based methacrylate oligomers containing imine functional group for stereolithography

Nuttapol Risangud[a,c], Jittima Mama [b], Piyarat Sungkhaphan[a], Puttipong Pananusorn [d], Orawan Termkunanon [b], Muhammad Sulthan Arkana[c], Supang Sripraphot[b], Tareerat Lertwimol[a], Somprasong Thongkham*[b]*

[a]National Metal and Materials Technology Center, National Science and Technology Development Agency, 111 Thailand Science Park, Phahonyothin Road, Klong Luang, Pathum Thani 12120, Thailand

[b]National Nanotechnology Center, National Science and Technology Development Agency, 111 Thailand Science Park, Paholyothin Rd., Klong 1, Klong Luang, Pathumthani 12120, Thailand

[c]Petroleum and Petrochemical College, Chulalongkorn University, Bangkok, 10330, Thailand

[d]Department of Materials Science and Engineering, School of Molecular Science and Engineering, Vidyasirimedhi Institute of Science and Technology (VISTEC), Wangchan, Rayong 21210, Thailand.

E-mail: Nuttapol Risangud (nuttapol.r@chula.ac.th) and Somprasong Thongkham (somprasong.tho@nanotec.or.th)

Contents

Table S1 Compositions of photosensitive resins composed of 2.9 wt% TPO and 0.1 wt% HQ **S3**

Figure S1 CAD models designed using Autodesk Netfabb software (Autodesk, San Rafael, USA) a) Cylindrical model, b-d) Gyroid model with 400, 600, and 800 µm wall thickness, respectively **S3**

Figure S2 ^{1}H NMR spectrum (DMSO-d_6, 400 MHz) of Monomer N2. **S4**

Figure S3 ^{1}H NMR spectrum (DMSO-d_6, 400 MHz) of FBMO. **S4**

Figure S4 SEC chromatogram of FBMO **S5**

Table S2 Curing depth of photosensitive resins at the exposure time of 40.0 seconds using a light source at the center of the vat* **S6**

Table S3 Size of 3D-printed specimens fabricated from different resin formulations using digital light processing (DLP) 3D printer. **S6**

Figure S5 Stress-strain curve of (a) all cylindrical specimens (i) CD-FBMO-50-PEG-50, (ii) CD-FBMO-50-TEG-50, (iii) CD-FBMO-70-PEG-30 (iv) CD-FBMO-70-TEG-30, (v) CD-FBMO-90-PEG-10, and (vi) CD- FBMO-90-TEG-10), and (b) of gyroid structures; (i) GR400- FBMO-90-PEG-10, (ii) GR600-FBMO-90-PEG-10, and (iii) GR800- FBMO-90-PEG-10. **S7**

Figure S6 Thermogravimetric curves of the post-cured specimens fabricated from different photosensitive resins. **S7**

Figure S7 SEM micrographs of post-cured gyroid structures fabricated from FBMO-90-PEG-10: (a) GR400-FBMO-90-PEG-10, (b) GR400-FBMO-90-PEG-10, and (c) GR400-FBMO-90-PEG-10. **S8**

Figure S8 FTIR spectra of post-cured gyroid structures: GR400-FBMO-90-PEG-10 and GR400-FBMO-90-PEG-10 after being treated with acetone/DI water (70/30 %v/v). **S8**

Table S1 Compositions of photosensitive resins composed of 2.9 wt% TPO and 0.1 wt% HQ

Photosensitive resin	FBMO (wt%)	PEGDMA (wt%)	TEGDMA (wt%)
FBMO-50-PEG-50	48.5	48.5	-
FBMO-50-TEG-50	48.5	-	48.5
FBMO-70-PEG-30	67.9	29.1	
FBMO-70-TEG-30	67.9		29.1
FBMO-90-PEG-10	87.3	9.7	
FBMO-90-TEG-10	87.3		9.7

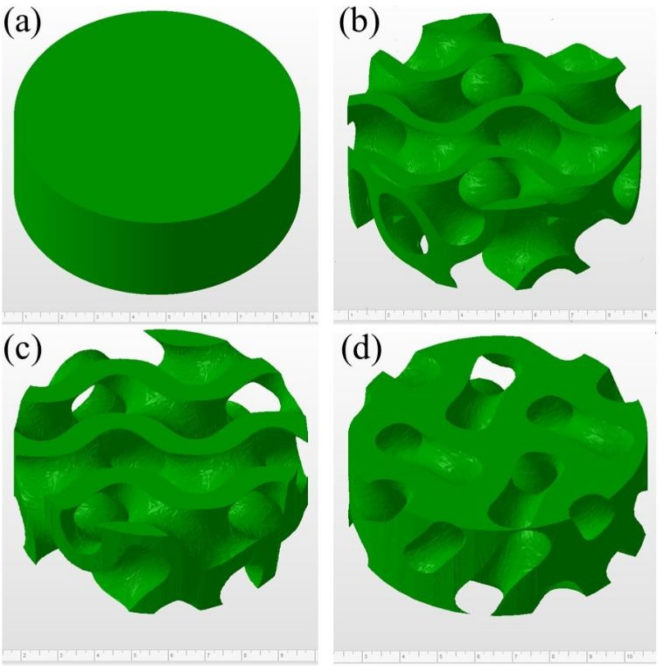

Figure S1 CAD models designed using Autodesk Netfabb software (Autodesk, San Rafael, USA) a) Cylindrical model, b-d) Gyroid model with 400, 600, and 800 μm wall thickness, respectively.

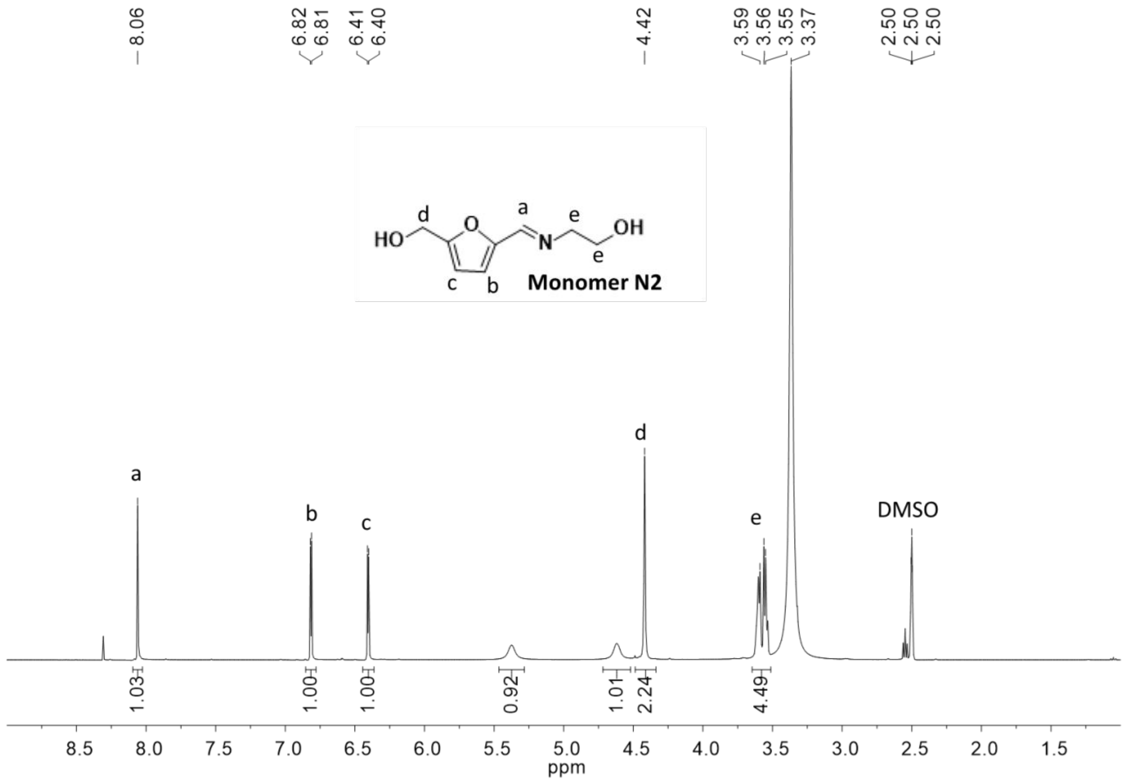

Figure S2 ^{1}H NMR spectrum (DMSO-d_6, 400 MHz) of Monomer N2.

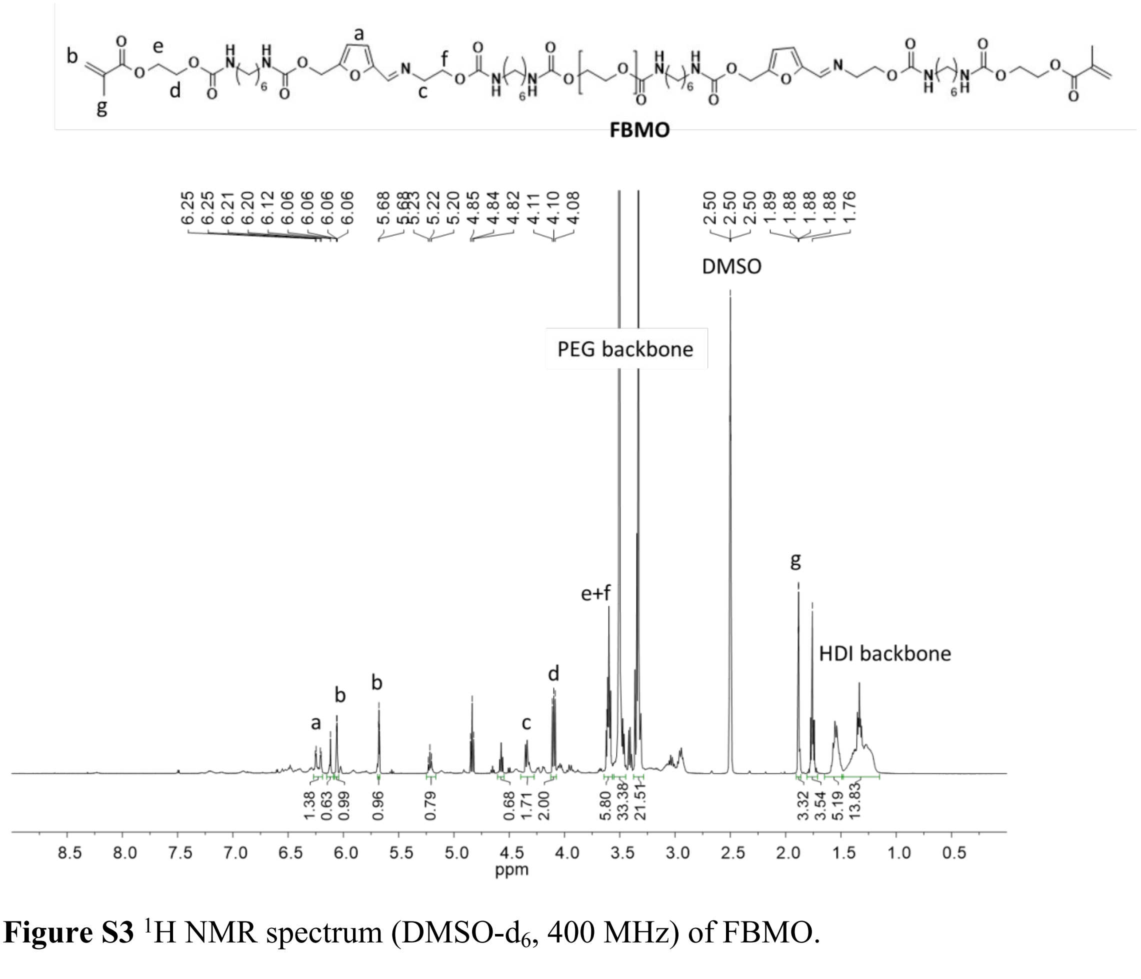

Figure S3 ^{1}H NMR spectrum (DMSO-d_6, 400 MHz) of FBMO.

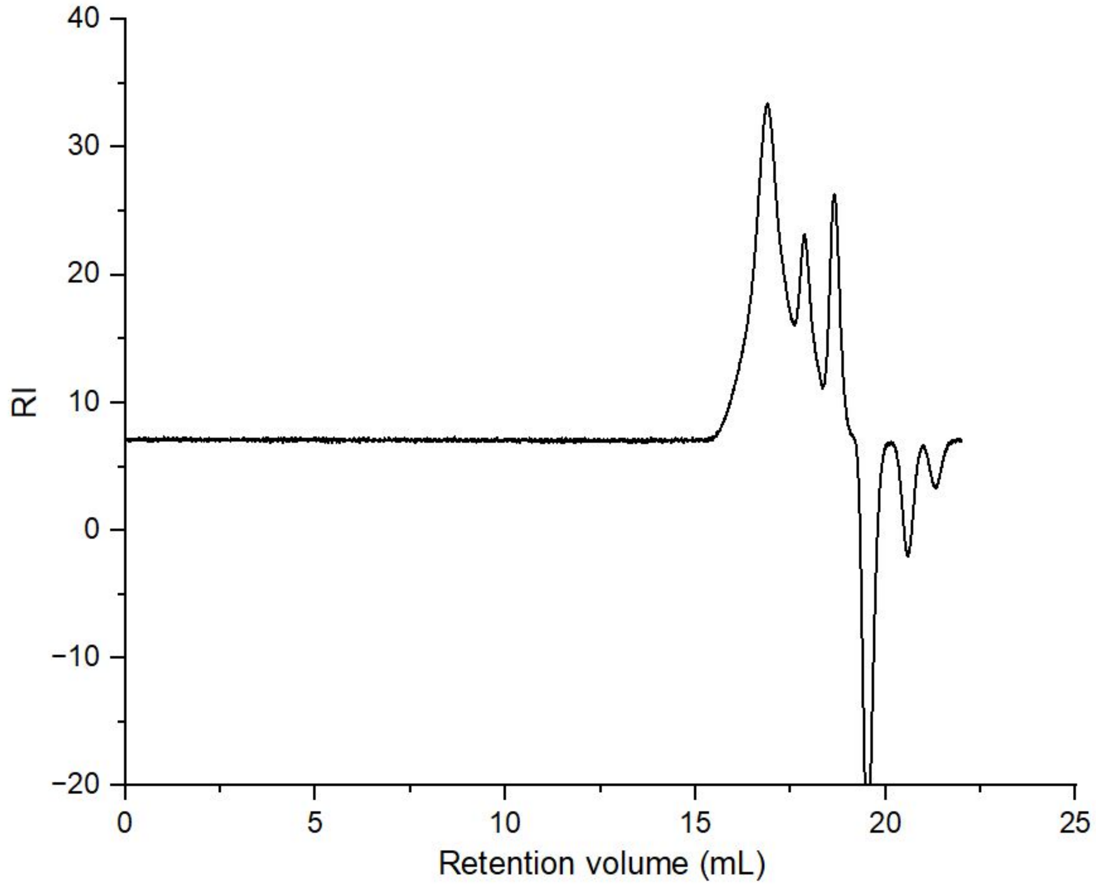

Figure S4 SEC chromatogram of FBMO

Table S2 Curing depth of photosensitive resins at the exposure time of 40.0 seconds using a light source at the center of the vat*

Photosensitive resin	Thickness of cured sample (mm)
FBMO-50-PEG-50	0.64±0.03[a]
FBMO-50-TEG-50	0.63±0.03[a]
FBMO-70-PEG-30	0.56±0.03[b]
FBMO-70-TEG-30	0.54±0.03[b]
FBMO-90-PEG-10	0.44±0.04[c]
FBMO-90-TEG-10	0.49±0.04[d]

*Experiments were conducted in quintuplicate, and the results are reported as the mean±SD. Different superscript lowercase letters indicate significant differences in the thickness of cured samples at $p < 0.05$.

Table S3 Size of 3D-printed specimens fabricated from different resin formulations using digital light processing (DLP) 3D printer.

3D-printed specimen	Size (mm)	
	Height	Diameter
CD-FBMO-50-PEG-50	3.12±0.01 (4%)	8.04±0.01 (1%)
CD-FBMO-50-TEG-50	3.12±0.03 (4%)	7.89±0.02 (-1%)
CD-FBMO-70-PEG-30	3.10±0.03 (3%)	8.08±0.01 (1%)
CD-FBMO-70-TEG-30	3.01±0.03 (3%)	7.89±0.09 (-1%)
CD-FBMO-90-PEG-10	3.08±0.02 (3%)	8.05±0.07 (1%)
CD-FBMO-90-TEG-10	3.10±0.02 (4%)	7.94±0.26 (-1%)
GR400- FBMO-90-PEG-10	3.06±0.02 (2%)	7.78±0.12 (-3%)
GR400- FBMO-90-TEG-10	3.07±0.03 (2%)	7.86±0.02 (-2%)
GR600- FBMO-90-PEG-10	3.07±0.04 (2%)	7.90±0.04 (-1%)
GR600- FBMO-90-TEG-10	2.98±0.07 (-1%)	7.81±0.07 (-2%)
GR800- FBMO-90-PEG-10	3.03±0.03 (1%)	7.87±0.02 (-2%)
GR800- FBMO-90-TEG-10	2.97±0.01 (-1%)	7.85±0.09 (-2%)

*Experiments were conducted in quintuplicate, and the results are reported as the mean±SD. The number in the bracket represents the percentage error of the resulting post-cured sample compared to the design; + and - indicate that the fabricated sample is bulkier and smaller than the design.

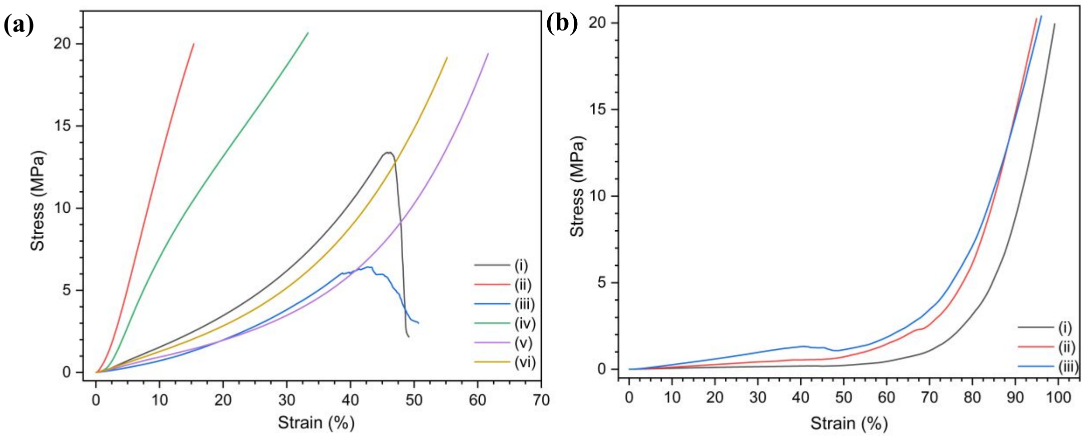

Figure S5 Stress-strain curve of (a) all cylindrical specimens (i) CD-FBMO-50-PEG-50, (ii) CD-FBMO-50-TEG-50, (iii) CD-FBMO-70-PEG-30 (iv) CD-FBMO-70-TEG-30, (v) CD-FBMO-90-PEG-10, and (vi) CD- FBMO-90-TEG-10), and (b) of gyroid structures; (i) GR400-FBMO-90-PEG-10, (ii) GR600- FBMO-90-PEG-10, and (iii) GR800- FBMO-90-PEG-10.

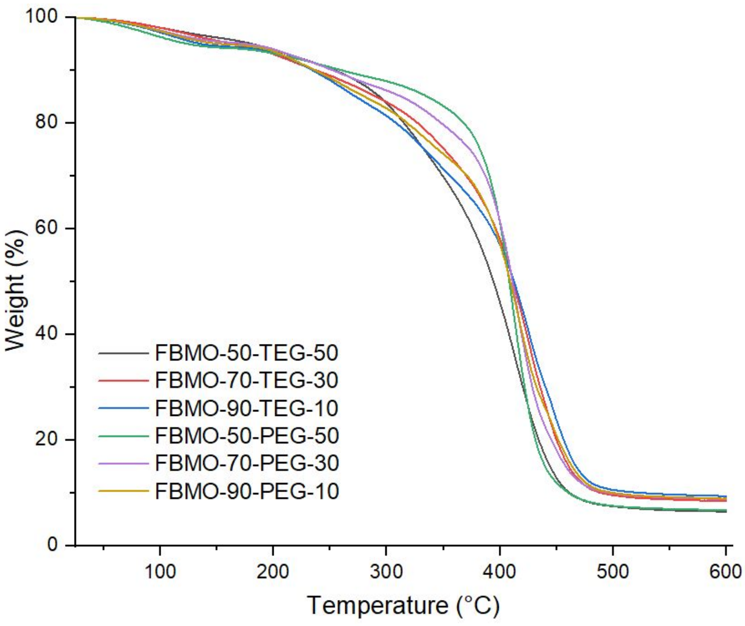

Figure S6 Thermogravimetric curves of the post-cured specimens fabricated from different photosensitive resins.

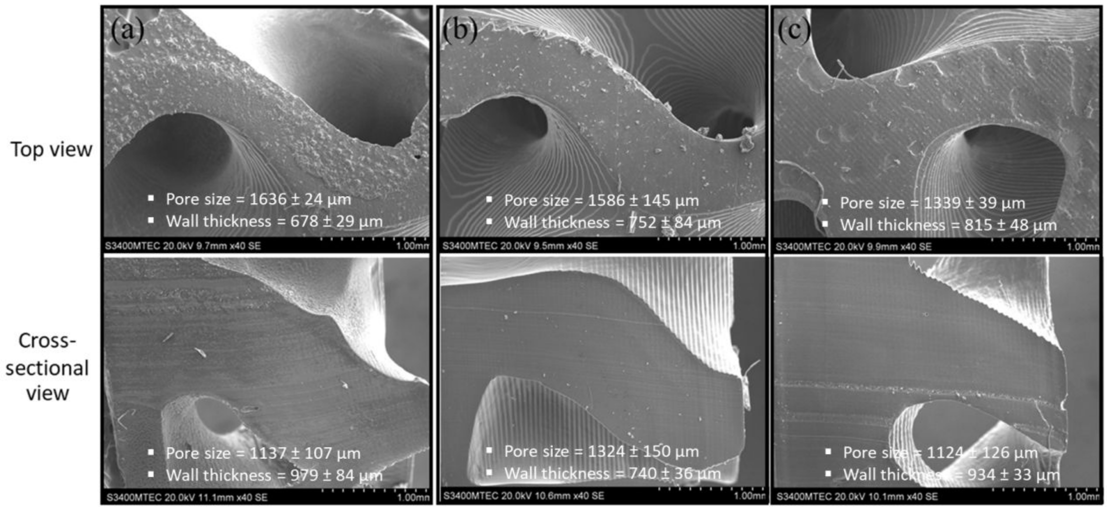

Figure S7 SEM micrographs of post-cured gyroid structures fabricated from FBMO-90-PEG-10: (a) GR400-FBMO-90-PEG-10, (b) GR400-FBMO-90-PEG-10, and (c) GR400-FBMO-90-PEG-10.

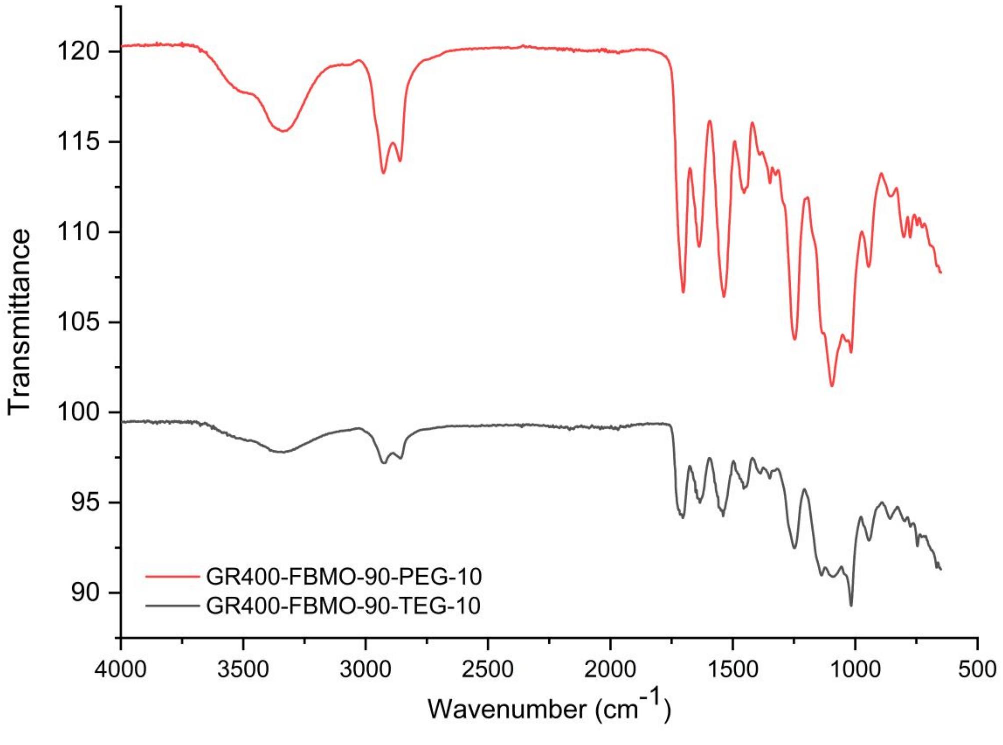

Figure S8 FTIR spectra of post-cured gyroid structures: GR400-FBMO-90-PEG-10 and GR400-FBMO-90-PEG-10 after being treated with acetone/DI water (70/30 %v/v).
